# Structural Studies of HHARI/UbcH7∼Ub Reveal Unique E2∼Ub Conformational Restriction by RBR RING1

**DOI:** 10.1016/j.str.2017.04.013

**Published:** 2017-06-06

**Authors:** Katja K. Dove, Jennifer L. Olszewski, Luigi Martino, David M. Duda, Xiaoli S. Wu, Darcie J. Miller, Katherine H. Reiter, Katrin Rittinger, Brenda A. Schulman, Rachel E. Klevit

**Affiliations:** 1 Department of Biochemistry, University of Washington, 1705 Northeast Pacific Street, Seattle, WA 98195, USA; 2 Department of Structural Biology, St. Jude Children's Research Hospital, 262 Danny Thomas Place, Memphis, TN 38105, USA; 3 Howard Hughes Medical Institute, St. Jude Children's Research Hospital, 262 Danny Thomas Place, Memphis, TN 38105, USA; 4 The Francis Crick Institute, 1 Midland Road, London NW1 1AT, UK

**Keywords:** RBR E3 ligase, RING E3 ligase, HHARI, ubiquitin, ubiquitin-conjugating enzyme, UbcH7, UBE2L3, UBA

## Abstract

RING-between-RING (RBR) E3s contain RING1 domains that are structurally similar yet mechanistically distinct from canonical RING domains. Both types of E3 bind E2∼ubiquitin (E2∼Ub) via their RINGs but canonical RING E3s promote closed E2∼Ub conformations required for direct Ub transfer from the E2 to substrate, while RBR RING1s promote open E2∼Ub to favor Ub transfer to the E3 active site. This different RING/E2∼Ub conformation determines its direct target, which for canonical RING E3s is typically a substrate or substrate-linked Ub, but is the E3 active-site cysteine in the case of RBR-type E3s. Here we show that a short extension of HHARI RING1, namely Zn^2+^-loop II, not present in any RING E3s, acts as a steric wedge to disrupt closed E2∼Ub, providing a structural explanation for the distinctive RING1-dependent conformational restriction mechanism utilized by RBR E3s.

## Highlights

• HHARI RING1 Zn^2+^-loop II extension disrupts closed conformation of E2∼Ubs
• A crystal structure shows open UbcH7∼Ub binding RING1 of auto-inhibited HHARI
• HHARI UBA-L domain has Ub binding properties

## Teaser

Dove et al. show that RING1 domains found in RING-between-RING (RBR) E3s, like HHARI, use Zn^2+^-loop II to disrupt closed E2∼Ub, resulting in open E2∼Ub that favor Ub transfer to the E3 active site. The work places the mechanism of RBR E3s-mediated Ub transfer on structural footing.

## Introduction

Post-translational modification of substrate with ubiquitin (Ub) requires the coordination of two types of enzymes: Ub-conjugating enzymes (E2s) and Ub ligases (E3s). While E3s are generally thought to bind substrates, substrate ubiquitination may be performed by either an E2 or an E3, depending on the type of E3 ligase involved. The large family of RING-type E3s bind a substrate and an E2∼Ub simultaneously and Ub transfer occurs from the E2 directly onto the substrate, most often a lysine (Lys) residue. In this case, the E2 transfers Ub via an aminolysis reaction and determines the type of Ub modification for a given substrate (Christensen et al., 2007, Kim et al., 2007, Rodrigo-Brenni and Morgan, 2007). In contrast, substrate ubiquitination by HECT (Homologous to E6AP C Terminus)-type E3s and by RING-between-RING (RBR) E3s is performed in two steps. First, the E3 binds a cognate E2∼Ub as a prelude to forming an obligatory E3∼Ub thioester intermediate. Second, Ub is transferred from the E3 to the substrate. In this case, the E2 transfers Ub via a transthiolation reaction and it is the E3 that transfers Ub to substrates and dictates the type of Ub modification independent of the E2 (Kim and Huibregtse, 2009, Kim et al., 2007, Wang et al., 2006, Wang and Pickart, 2005). Among the several dozen human E2s, UbcH7 is unique in its ability to react solely via transthiolation reactions, making it an RBR/HECT-only E2 (Wenzel et al., 2011). However, many E2s including the well-known UbcH5 family are known to work with both types of E3s, raising the question of how they discriminate between performing aminolysis reactions when paired with a RING E3 and transthiolation reactions when paired with an RBR or HECT E3.

**Figure 1:**
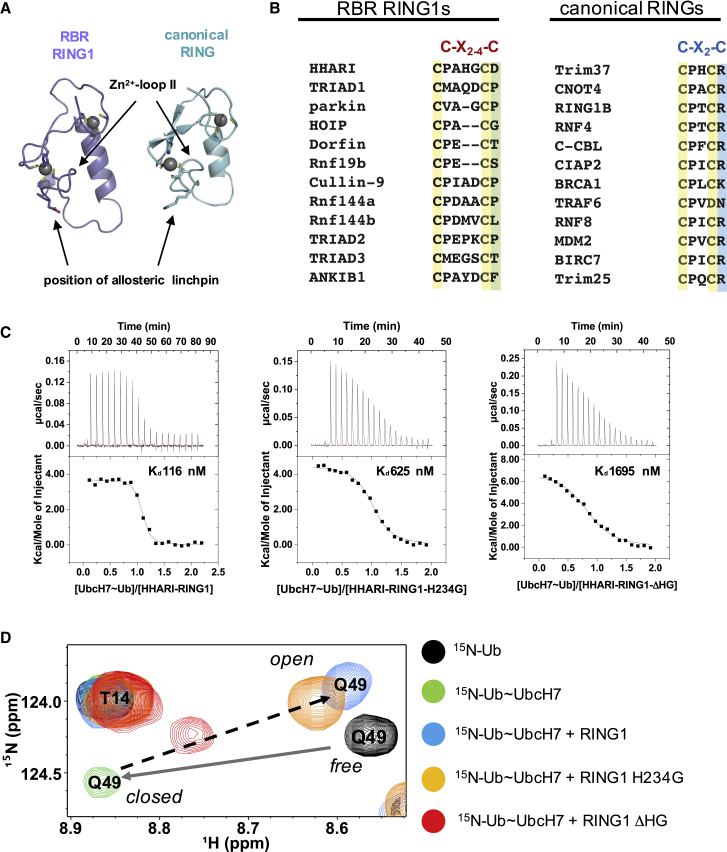
RING1 Domains Have an Extended Zn^2+^-Loop II (A) Comparison of RING1 and RING domains. The structures of RING1 of HHARI (slate, PDB: 4KC9) and the RING domain of BRCA1 (cyan, PDB: 1JM7) illustrate the similar overall topologies of the two related domains. Zn^2+^-loop II and the position of the allosteric linchpin are indicated with arrows. The residue at the position of the allosteric linchpin and the residue at Zn^2+^ coordination are represented as sticks. (B) KALIGN sequence alignment of Zn^2+^-loop II segments of RBR RING1 (left) and canonical RING domains (right). The seventh and eighth zinc-coordinating Cys residues for each domain are highlighted in yellow. The allosteric linchpin found in >50% of canonical RINGs is highlighted in blue; there are no functionally analogous residues at this position in RING1s (green). Zn^2+^-loop II is invariably two residues long in canonical RING domains (C-X_2_-C), but varies in length in RBR RING1s (C-X_2-4_-C). (C) Calorimetric titrations of UbcH7∼Ub and HHARI RING1 wild-type (left), H234G (middle), and the two-residue loop deletion mutant ΔHG (right). Both the raw data and normalized binding curves are shown. Calorimetric titrations with unconjugated UbcH7 are shown in Figure S1B. (D) The length of Zn^2+^-loop II is largely responsible for the ability of HHARI RING1 to promote UbcH7∼Ub open states. A signature of closed states populated by UbcH7∼Ub in the absence of E3 is a large chemical-shift perturbation of Gln49 ^15^N-Ub (black versus green spectrum; gray arrow). Binding of HHARI RING1 to UbcH7∼Ub reverses the chemical shift of Gln49 (green-to-blue spectrum; black arrow), indicative of disruption of closed UbcH7∼Ub (Dove et al., 2016). Shortening of HHARI RING1 Zn^2+^-loop II by two residues (ΔHG) reduces the ability of HHARI RING1 to promote open UbcH7∼Ub (red spectrum), but RING1 harboring a mutation of His to Gly still promotes open states of UbcH7∼Ub (orange spectrum). See also Figures S1 and S2.

RBR E3s share structural features with RING-type E3s in that they contain an E2-binding RING domain (called “RING1” in RBRs) (Eisenhaber et al., 2007). Structures of RBR RING1 domains from Parkin, HHARI, HOIP, and RNF144 confirm that they are highly similar to canonical RINGs (Figure 1A) (Duda et al., 2013, Lechtenberg et al., 2016, Miyamoto et al., 2004, Riley et al., 2013, Trempe et al., 2013, Wauer and Komander, 2013). However, there is growing evidence that canonical RINGs and RBR RING1s are mechanistically distinct. Specifically, canonical RINGs promote closed conformations of E2∼Ubs that have enhanced reactivity toward Lys, while RBR RING1s promote open E2∼Ub serving to inhibit aminolysis reactions, thereby ensuring a transthiolation reaction to generate the E3∼Ub conjugate (Dou et al., 2012, Dove et al., 2016, Plechanovova et al., 2012, Pruneda et al., 2012). These distinct strategies used by structurally similar domains allow E3s to modulate E2 reactivity to specific needs.

Consistent with a two-step mechanism, RBR E3s display mechanistic parallels to HECT type-E3s, with a distinct active-site-containing domain (Wenzel et al., 2011), although the catalytic domain in RBRs lacks structural homology with that found in HECT E3s. In available structures of a HECT- and an RBR-type E3 bound to UbcH5∼Ub, the conjugate is open and Ub contacts the catalytic domain, the HECT C lobe or RING2, respectively (Kamadurai et al., 2009, Lechtenberg et al., 2016). A simple explanation for these observations is that the Ub/catalytic domain interaction promotes the open E2∼Ub when bound to these E3s. However, we recently reported that isolated RING1 domains from HHARI and RNF144 are sufficient to disrupt closed states of E2∼Ubs without detectable contact with the Ub, as evidenced by nuclear magnetic resonance (NMR) studies (Dove et al., 2016). This implies that these RING1 domains have intrinsic features that somehow promote open E2∼Ub states while highly similar canonical RING domains promote closed E2∼Ub states.

Here we address this conundrum for the RBR E3 HHARI and UbcH7∼Ub conjugate. NMR and mutagenesis reveal that the second Zn^2+^ loop (Zn^2+^-loop II) in RING1 is largely responsible for disrupting closed UbcH7∼Ub states. In a new crystal structure of an HHARI/UbcH7∼Ub complex, the RING1-bound E2∼Ub conjugate is in an open state and the Ub does not contact the catalytic RING2 domain. Zn^2+^-loop II of RING1 contacts UbcH7 on a surface that would conflict with Ub in a closed E2∼Ub conformation. The structure also verifies that E2∼Ub binding does not release HHARI from its auto-inhibited conformation, implying that additional events must occur to release the E3 into its active form. Relevant to the reported activation of HHARI by binding to neddylated Cullins, we also extend previous results (Kelsall et al., 2013) to demonstrate that HHARI can bind free NEDD8 and free Ub through its UBA-like domain.

## Results

### Zn^2+^-Loop II of HHARI RING1 Promotes Open UbcH7∼Ub Conformations

To understand how RING1s promote opening of E2∼Ubs, we compared primary sequence alignments of RING1s and canonical RING domains. RING and RING1 domains are defined by a small number of conserved residues, most of which are required for coordinating two Zn^2+^ ions. Most canonical RINGs also share elements that stimulate E2∼Ub activity including a conserved “linchpin” residue that RBR RING1s appear to lack (Figure 1B) (Das et al., 2009, Dou et al., 2012, Dou et al., 2013, Koliopoulos et al., 2016, Plechanovova et al., 2012, Pruneda et al., 2012, Scott et al., 2014). Lack of a linchpin could explain why RING1s do not promote closed E2∼Ubs, but does not explain how they actively disfavor closed E2∼Ubs (Dove et al., 2016). A unique feature of RING1s is an extension of Zn^2+^-loop II, a loop involved in E2 binding (Lechtenberg et al., 2016, Metzger et al., 2014, Spratt et al., 2014). While the last two Zn^2+^-coordinating cysteine (Cys) residues are consistently separated by exactly two residues in canonical RINGs (C_7th_-X-X-C_8th_), there are up to four residues in RING1s (Figure 1B) (Spratt et al., 2014). The Zn^2+^-loop II of canonical RINGs typically contacts E2s via loop 7 near the E2 crossover helix, a surface that contacts Ub in closed E2∼Ubs (Figure S1A) (Dou et al., 2012, Dou et al., 2013, Plechanovova et al., 2012, Pruneda et al., 2012). However, the HHARI RING1 binding surface on the E2 UbcH7 includes the crossover helix, suggesting a potential overlap with the Ub contact site of closed E2∼Ubs (Dove et al., 2016). Putting these observations together, we hypothesized that the longer Zn^2+^-loop II of HHARI RING1 (C_7th_-P-A-H-G-C_8th_) might sterically impede closed states of E2∼Ubs.

To test our hypothesis, we designed two HHARI RING1 mutants and assessed their ability to disrupt closed UbcH7∼Ubs. In one mutant, two “extra” loop residues, His234 and Gly235, were deleted to create a more canonical two-residue loop (“ΔHG”) and in the other, the side chain of His234 was trimmed to Gly (H234G) to remove a bulky side chain. Binding affinities measured by isothermal titration calorimetry (ITC) reveal that HHARI RING1 binds UbcH7∼Ub with high affinity (K_D_ 116 nM; Figure 1C and Table 1). Although the mutations reduce the affinity for UbcH7∼Ub, these remain relatively tight (K_D_ of 625 nM and 1.7 μM for H234G-RING1 and ΔHG-RING1, respectively; Figures 1C and S1B; Table 1). The slower auto-ubiquitination observed in multiple turnover assays for the ΔHG mutation in the context of the HHARI RBR module (GST-HHARI^RBR^) at UbcH7∼Ub concentrations below the mutant K_D_ is largely overcome at higher concentrations of E2 (Figure S1C). We therefore characterized each mutants' ability to disrupt closed UbcH7∼Ubs conformations.

**Table 1:** ITC Data for Interactions between HHARI RING1 and Its Mutants with UbcH7, UbcH7∼Ub, UbcH5a, and UbcH5a∼Ub

Interactions	n	K_D_ (nM)	ΔH (kcal/mol)	−TΔS (kcal/mol)	ΔG (kcal/mol)
RING1/UbcH7	1.0 ± 0.1	294 ± 25	4.3 ± 0.1	−12.7 ± 0.8	−8.4 ± 0.7
RING1/UbcH7∼Ub	1.0 ± 0.1	116 ± 34	3.7 ± 0.1	−12.7 ± 2.7	−8.9 ± 2.6
RING1-ΔHG/UbcH7	0.8 ± 0.2	2,380 ± 300	4.2 ± 0.9	−11.7 ± 1.8	−7.5 ± 0.9
RING1-ΔHG/UbcH7∼Ub	0.9 ± 0.1	1,695 ± 152	6.9 ± 0.9	−14.6 ± 1.6	−7.7 ± 0.7
RING1-H234G/UbcH7	0.8 ± 0.2	1,000 ± 102	7.2 ± 0.8	−15.2 ± 1.6	−8.0 ± 0.8
RING1-H234G/UbcH7∼Ub	1.0 ± 0.1	625 ± 79	4.5 ± 0.5	−12.8 ± 1.5	−8.3 ± 1.0
RING1/UbcH5a	no binding detected				
RING1/UbcH5a∼Ub	no binding detected				

To assess whether the above RING1 mutants affect the open/closed status of UbcH7∼Ub, we focused on key NMR resonances in the Ub spectrum that report on these states, using a stable oxyester mimic (active-site Cys-to-Ser mutant) of UbcH7∼Ub as described previously (Dove et al., 2016). The NMR spectrum of ^15^N-Ub within UbcH7∼Ub exhibits chemical shifts that are hallmarks of the closed state (Dove et al., 2016). For example, the Q49-Ub NH resonance undergoes a large chemical shift relative to its position in free ^15^N-Ub upon conjugation to UbcH7 and shifts back toward its position in free ^15^N-Ub upon HHARI RING1 binding to UbcH7∼Ub (Figure 1D, from black to green to blue, respectively). The observations indicate that UbcH7∼Ub is predominantly in closed states on its own and is predominantly in open conformations when bound to RING1 (Dove et al., 2016). The Q49-Ub resonance falls along a trajectory defined by the resonances of unbound UbcH7∼Ub (green) and bound to wild-type (WT) HHARI RING1 (blue) when bound to the mutant HHARI RING1s (ΔHG and H234G; Figure 1D, red and orange). The most parsimonious explanation is that the RING1 mutants affect the fractional populations of closed and open UbcH7∼Ub states. Specifically, the Q49-Ub resonance remains closer to the “predominantly closed-state” position (Figure 1D, red) when bound to the loop deletion mutant while it still shifts almost to the WT-RING1-bound position when bound to H234G-RING1 (Figure 1D, orange). Therefore, RING1 that lacks the His234 side chain can disrupt closed E2∼Ubs while RING1 with a shortened loop has diminished ability to perform this function. Other Ub resonances that undergo large chemical-shift changes in open versus closed UbcH7∼Ub states show behaviors similar to those of the Q49 peak (Figure S2A). The observations cannot be explained by decreased binding affinities of UbcH7∼Ub to mutant RING1s, as NMR experiments were performed under saturating conditions. Altogether, the results indicate that the length of Zn^2+^-loop II rather than the nature of its side chains is responsible for the ability of HHARI RING1 to promote open UbcH7∼Ubs. Consistent with this conclusion, RNF144 RING1 also disrupts closed UbcH7∼Ub, yet it contains an Ala at the position analogous to HHARI His234 (Figure 1B) (Dove et al., 2016). The loop deletion mutant of HHARI does elicit a small shift away from the closed state position, indicating that there may be contributions from other portions of RING1. Nevertheless, the data reveal that the extension of Zn^2+^-loop II plays a major role in HHARI's ability to promote open E2∼Ubs.

Previous studies have established that open states of E2∼Ub have low reactivity in aminolysis reactions and that canonical RING domains activate E2∼Ub conjugates to transfer Ub to Lys residues by promoting closed E2∼Ub conjugates (Dou et al., 2012, Plechanovova et al., 2012, Pruneda et al., 2012). A recent study showed that RBR RING1 domains disrupt highly Lys-reactive closed states to inhibit aminolysis reactions of E2s when they are bound to RBR E3s, ensuring that transfer occurs via the RING2 active-site Cys (Dove et al., 2016). This modulation of reactivity allows Lys-reactive E2s to function with canonical RING and RBR E3s. However, HHARI RING1 also disrupts closed UbcH7∼Ubs, even though UbcH7 is intrinsically unreactive toward Lys (Wenzel et al., 2011). We therefore wondered whether open UbcH7∼Ub states might be more favorable for Ub transfer to the active-site Cys of RBR E3s. The hydrophobic surface of Ub has been shown to bind RING2, and mutations to residues on either surface inhibit formation of the E3∼Ub intermediate with HHARI and HOIP (Dove et al., 2016, Lechtenberg et al., 2016, Scott et al., 2016). However, that surface of Ub is sequestered in closed UbcH7∼Ub conformations (Dove et al., 2016). We assessed the ability of UbcH7∼Ub to generate the E3∼Ub conjugate when bound to WT- and ΔHG-HHARI^RBR^ to see whether promoting open E2∼Ub has a direct effect on the transthiolation reaction. The reaction and subsequent E3∼Ub discharge are both rapid, so experiments were performed in the background of a RING2 mutation (H359A) that allows the HHARI∼Ub intermediate to be detected (Figure S2B) (Duda et al., 2013). Although we are unable to quantify rates of reaction, it is clear that the loop deletion mutant can form an E3∼Ub intermediate, indicating that UbcH7∼Ub visits open states frequently enough for productive transthiolation events in the context of our assay (Figure S2B). The result is consistent with the NMR spectrum that shows that ΔHG-HHARI RING1 produces an intermediate population distribution of open and closed UbcH7∼Ub states (Figure 1D). Taken together, the results shown here and in previous studies lead us to conclude that while the ability of canonical RINGs to promote closed E2∼Ubs is a positive determinant for E2∼Ub activation to transfer to Lys, the ability of RING1s to inhibit closed E2∼Ub states is a negative determinant that inhibits transfer to Lys and ensures that transfer occurs through the RBR active site. Although the negative feature is not critical for an RBR when it reacts with UbcH7, which cannot transfer Ub directly to Lys, many RBRs including HHARI are also known to act with Lys-reactive E2s, including UbcH5, Ubc13, and Ube2k (Dove et al., 2016, Fiesel et al., 2014, Haddad et al., 2013, Kirisako et al., 2006, Lechtenberg et al., 2016, Lim et al., 2013, Stieglitz et al., 2013, Wenzel et al., 2011).

### Structure of HHARI Bound to UbcH7∼Ub

**Figure 2:**
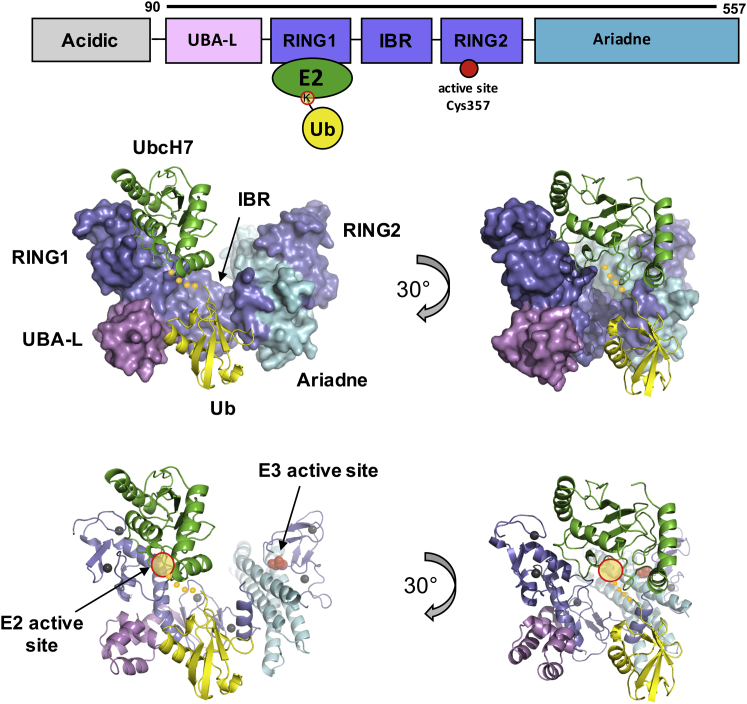
Structure of HHARI/UbcH7∼Ub (Top) The domain architecture of HHARI. The construct used for crystallography is denoted by the black bar. The HHARI active-site Cys is marked with a full red circle. The active site (C86K) of UbcH7 is marked with a yellow circle (red outline). (Bottom) HHARI is shown in surface (upper) and cartoon (lower) representation, with same domain coloring as in the top panel. UbcH7∼Ub is shown in cartoon representation (UbcH7 in green, Ub in yellow). Auto-inhibited HHARI binds to UbcH7∼Ub in an open conformation, and no contacts are observed between Ub and its cognate HHARI (as emphasized by 30° rotation panels on right). The UbcH7 active-site C86K (yellow circle, red outline) and HHARI active site (red spheres) are indicated. Yellow dots represent the C-terminus of Ub for which there is no observable density. The HHARI active-site Cys on RING2 is not visible in the surface representation (left) as it is occluded by the Ariadne domain (cyan). See also Figure S3.

To obtain a more detailed view of the interactions between HHARI and UbcH7∼Ub, we determined a 3.2-Å resolution crystal structure of HHARI (residues 90–557; for simplicity referred to as HHARI, Figure 2, top; Table 2) bound to UbcH7∼Ub (Figure 2) using the active site Cys-to-Lys mutant of UbcH7 to increase conjugate stability (referred to as UbcH7∼Ub, Figure 2, top). The asymmetric unit contains two copies of HHARI each bound to UbcH7∼Ub. Weak density was observed for the two Ub moieties, with only one placed into the final model. The interface between the two copies of the complex is composed of two RING1s and two UbcH7 molecules, with one interface utilizing the N term (canonical RING binding) and the other interface the C term of UbcH7 (non-canonical interaction). Size-exclusion chromatography coupled with multi-angle light scattering (SEC-MALS) analysis of HHARI/UbcH7 in solution revealed a single sharp elution peak and an observed molecular weight of 80.5 kDa (predicted molecular weight 81.98 kDa; Figure S3A), consistent with a complex composed of one copy each of HHARI and UbcH7, indicating that the putative hetero-tetramer in the crystal is not populated in solution.

**Table 2:** Data Collection and Refinement Statistics

	HHARI/UbcH7∼Ub (Zn-SAD)	HHARI/UbcH7∼Ub (Native)
**Data Collection**		

Space group	*C*2	*C*2
Unit cell *a*, *b*, *c* (Å), α, β, γ (°)	183.71, 75.82, 147.61, 90, 107.15, 90	184.57, 76.79, 147.72, 90, 107.33, 90
Resolution (Å)	141.04–3.56 (3.85–3.56)^a^	48.45–3.24 (3.45–3.24)
Completeness (%)	96.4 (97.9)	98.9 (96.0)
No. of reflections (unique)	65,236 (22,642)	183,340 (31,387)
Redundancy	2.9 (2.9)	5.8 (5.9)
*R*_sym_ (%)	7.5 (56.1)	9.3 (88.3)
*R*_meas_ (%)	9.1 (68.3)	10.2 (96.6)
_CC1/2_	1.00 (0.72)	1.00 (0.76)
*I*/σ*I*	13.3 (2.1)	13.1 (1.9)

**Refinement**		

Resolution range (Å)		48.45–3.24
*R*_work_/*R*_free_		22.7/27.9
No. of atoms protein/Zn		9,166/12
RMSD bond lengths (Å)		0.009
RMSD bond angles (°)		1.4
*B* factor (Å^2^) protein/Zn		116.6/122.4

RMSD, root-mean-square deviation.

a Values in parentheses represent the highest-resolution shell and preceding values are for all data.

Two features are immediately apparent in the structure of the complex (Figure 2). First, although there is an E2∼Ub bound to RING1, the E3 is in an auto-inhibited conformation similar to previous structures of HHARI (PDB: 4KC9 and 4KBL; Duda et al., 2013). Second, UbcH7∼Ub is in an open conformation consistent with earlier conclusions that HHARI RING1 promotes open states of UbcH7∼Ub (Dove et al., 2016). Though weak, the electron density observed for the Ub moiety does not contact its cognate HHARI partner. This contrasts with available structures of UbcH5∼Ub bound to active thioester-forming E3s: the HECT domain from NEDD4L (PDB: 3JVZ and 3JW0) where the HECT C lobe makes intimate contact with a hydrophobic surface of Ub (Kamadurai et al., 2009), and an “active” form of HOIP RBR-LDD that interacts with the Ub moiety of UbcH5∼Ub (PDB: 5EDV; Lechtenberg et al., 2016).

### HHARI Is Auto-inhibited Even When Bound to UbcH7∼Ub

An emerging property of some RBR E3s is that their activity is kept in check by adoption of auto-inhibited states. Full-length HHARI, as well as the construct used in this study, does not exhibit detectable E3 ligase activity in vitro (Duda et al., 2013, Scott et al., 2016). From its auto-inhibited state, HHARI can bind UbcH7 or its conjugate, but this binding does not relieve the inhibition (Duda et al., 2013, Scott et al., 2016). Consistent with this observation, HHARI is still in an auto-inhibited conformation in the structure of the HHARI/UbcH7∼Ub complex reported here: the active site in RING2 is far from the UbcH7 active site and occluded by the Ariadne domain (Figure 2). Comparison with a structure of the HOIP RBR-LDD module bound to an E2∼Ub (PDB: 5EDV) reveals some similarities, but also striking differences (Figure S3B) (Lechtenberg et al., 2016, Smit et al., 2012, Stieglitz et al., 2012). As in HHARI/UbcH7∼Ub, HOIP RING1 binds UbcH5∼Ub in an open conformation. In the latter case, the E3 makes substantial contacts to the ∼Ub (Ub conjugated to the E2) via the C-terminal helix of RING1, the in-between RING (IBR), and RING2 (Figure S3B). The ∼Ub/RING2 contacts observed involve a non-cognate molecule of HOIP in the crystal, but are similar to those seen in a structure of an E3∼Ub mimic of the HOIP RING2-LDD module (PDB: 4LJO and 4LJP; Stieglitz et al., 2013). Together, these observations suggest that Ub/RING2 interactions play a role both prior to and after the transthiolation step from E2∼Ub to E3∼Ub and that the contact observed in *trans* in the HOIP/E2∼Ub crystal is a proxy for a mechanistically relevant interaction (Lechtenberg et al., 2016, Stieglitz et al., 2013). Two recent studies have concluded that similar Ub/RING2 contacts are important in the formation of the HHARI∼Ub intermediate (Dove et al., 2016, Scott et al., 2016). However, structural details regarding the large conformational change required to bring E2∼Ub and RING2 in proximity remain to be defined.

### Unique Interactions between HHARI RING1 and UbcH7

Although UbcH7 can bind to canonical RINGs, it has thus far been shown to transfer Ub only to Cys residues, and therefore its known RING-dependent activity is restricted to RBR E3s (Brzovic et al., 2003, Christensen et al., 2007, Wenzel et al., 2011, Zheng et al., 2000). Comparison of functional E2/RING pairs, mostly containing the E2 UbcH5, with the non-functional C-cbl RING/UbcH7 (PDB: 1FBV) structure confirms a similar binding mode (Zheng et al., 2000).

**Figure 3:**
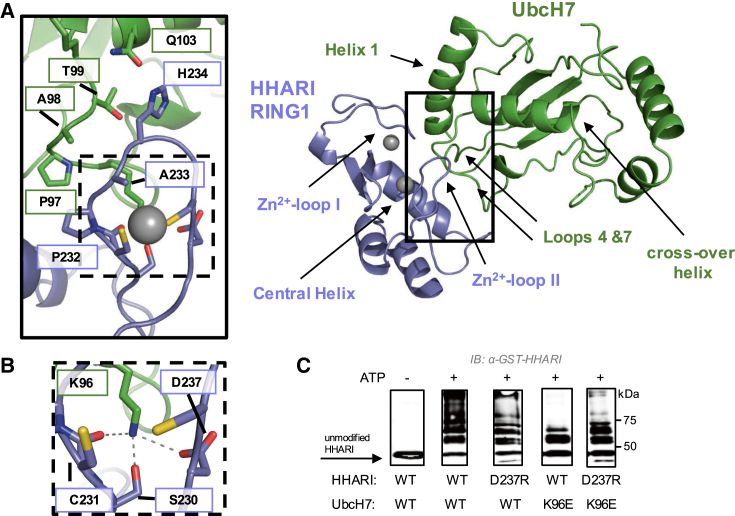
Unique Features of the RING1/UbcH7 Interface (A) UbcH7 loop 7 and the beginning of the crossover helix form extensive contacts with HHARI Zn^2+^-loop II residues Ser230-Asp237. UbcH7 crossover helix residue Gln103 is within hydrogen-bound distance of RING1 His234. Important structural features of RING1 domains and the E2 are labeled with arrows (right). (B) UbcH7 Lys96 forms hydrogen bonds to RING1 Zn^2+^-loop II residues Ser230, Cys231, and Asp237. Zn^2+^ is not shown for clarity. (C) Auto-ubiquitination assays with UbcH7 and GST-HHARI^RBR^, which acts as both E3 and proxy substrate. This construct lacks the Ariadne domain and is therefore active (Duda et al., 2013). Single charge-swap mutations in RING1 (D237R) or UbcH7 (K96E) reduced auto-ubiquitination activity when combined with either wild-type (WT) UbcH7 or WT HHARI, respectively. When D237R-HHARI is combined with K96E-UbcH7, higher levels of activity are observed compared with K96E-UbcH7 and WT HHARI, providing further evidence that RING1 Asp237 and UbcH7 Lys96 form a salt bridge at the RING1/UbcH7 interface. Reactions were quenched after 15 min post ATP addition with SDS-PAGE load dye, and products were visualized by western blotting against GST. Image was cropped to display 0 min (lane 1) and 15 min time points only (lanes 2–5). See also Figures S4 and S5.

The overall architecture of the RING1/UbcH7 interface is similar to those in other RING/E2 pairs (Brown et al., 2014, Brzovic et al., 2003, DaRosa et al., 2015, Dou et al., 2012, Koliopoulos et al., 2016, Lechtenberg et al., 2016, Li et al., 2015, Plechanovova et al., 2012, Pruneda et al., 2012, Scott et al., 2014, Xu et al., 2008, Zheng et al., 2000). UbcH7 residues from loops 4 and 7 and helix 1 and HHARI RING1 residues from both Zn^2+^ loops and the central helix make canonical E2/RING interactions (Figures 3A and S1A). Residues that are in common between UbcH7 and UbcH5 in the RING-binding regions make similar contacts. For example, UbcH7 loop-4 residue Phe63 contacts HHARI RING1 residues Ile188 in Zn^2+^-loop I and Tyr215 in the central helix (Figure S4A). The analogous Phe62 in UbcH5 contacts canonical RINGs via a conserved Ile residue that is analogous to HHARI Ile188. Likewise, hydrophobic residues from the central helix of RINGs are involved in E2 interactions. Substitution at any of these three positions with alanine results in a substantial decrease in HHARI^RBR^ auto-ubiquitination activity (Figure S4B; Duda et al., 2013, Scott et al., 2016), and the double mutant I188A/Y215A-RING1 exhibits a complete loss of detectable ligase activity under the same conditions (Figure S4C).

HHARI functions with both UbcH7 and UbcH5 but has markedly higher affinity for UbcH7 than for UbcH5 (Table 1; Figures S1B and S5A) (Wenzel et al., 2011). Interestingly, we noted that loop 4 is well conserved while loop-7 residues vary between UbcH7 and UbcH5 (Figure S5B). There are extensive contacts between RING1 Zn^2+^-loop II and UbcH7 loop 7, with virtually every residue of HHARI's loop extending from residue 230–237 in contact with UbcH7's loop 7 and the beginning of the crossover helix (Figure 3A). Importantly, the previously discussed HHARI His234 in Zn^2+^-loop II contacts UbcH7 residue Gln103 (Figure 3A). Substitution of His234 with a Gly decreases the affinity of HHARI RING1 for UbcH7 from ∼300 nM to 1,000 nM (Table 1). This decrease corresponds to a ΔΔG of 0.4 kcal/mol, consistent with the loss of an H bond (Sheu et al., 2003).

Another UbcH7-specific interaction involves Lys96 in loop 7 of UbcH7, in the position normally held by a serine in the highly conserved “WSPAL” motif of numerous E2s including UbcH5 (Figure S5B). The structure reveals an extensive hydrogen-bond network emanating from Lys96 to three RING1 Zn^2+^-loop II residues (Ser230, Cys231, and Asp237; Figure 3B). HHARI residue Asp237 is in what would be the linchpin position of RING1, based on primary sequence. A charge-swap mutant of Lys96, K96E-UbcH7, shows greatly diminished activity in auto-ubiquitination of HHARI^RBR^ (Figure 3C). Similarly, a charge-swap mutant of HHARI residue Asp237, D237R-HHARI^RBR^, has decreased activity relative to the WT-HHARI^RBR^ (Figure 3C). As predicted from the structure, mutation of the Lys that interacts with three groups shows a greater reduction in activity than mutation of RING1 D237 that disrupts a single contact. Notably, when the charge-swapped E2 and E3 mutants are combined in the same assay, increased activity is observed relative to the activity of either mutant paired with the WT partner (Figure 3C). The results confirm that an ionic interaction between Lys96 in UbcH7 and Asp237 in HHARI inferred from the crystal structure contributes to the formation of a functional UbcH7/HHARI RING1 complex.

Our NMR results indicate that Zn^2+^-loop II of HHARI is largely responsible for disfavoring the closed state of UbcH7∼Ub (Figure 1D). Previous NMR mapping revealed that the HHARI RING1-binding surface extends onto the crossover helix of UbcH7 and includes Gln103 (Dove et al., 2016). Consistent with those observations, the tip of Zn^2+^-loop II reaches past UbcH7's loop 7 to contact Gln103 on the E2 crossover helix, as mentioned above (Figure 3A). The analogous Lys101 UbcH5 side chain contacts Ub in the closed state of UbcH5∼Ub when bound to canonical RING domains (Dou et al., 2012, Koliopoulos et al., 2016, Plechanovova et al., 2012). Taken together, these observations suggest that RING1 Zn^2+^-loop II may conflict sterically with the position of Ub in closed E2∼Ub. To assess this possibility, we built a model of RING1/UbcH7∼Ub with Ub in the closed state by superimposing the structure of closed UbcH5∼Ub from the complex with BIRC7 (PDB: 4AUQ; Figure S5C). In the model, the tip of Zn^2+^-loop II of HHARI RING1 clashes with Ub in the closed state of E2∼Ub, providing a structural explanation for results presented in Figure 1.

### The HHARI UBA-L Domain Binds to Ubiquitin and NEDD8

**Figure 4:**
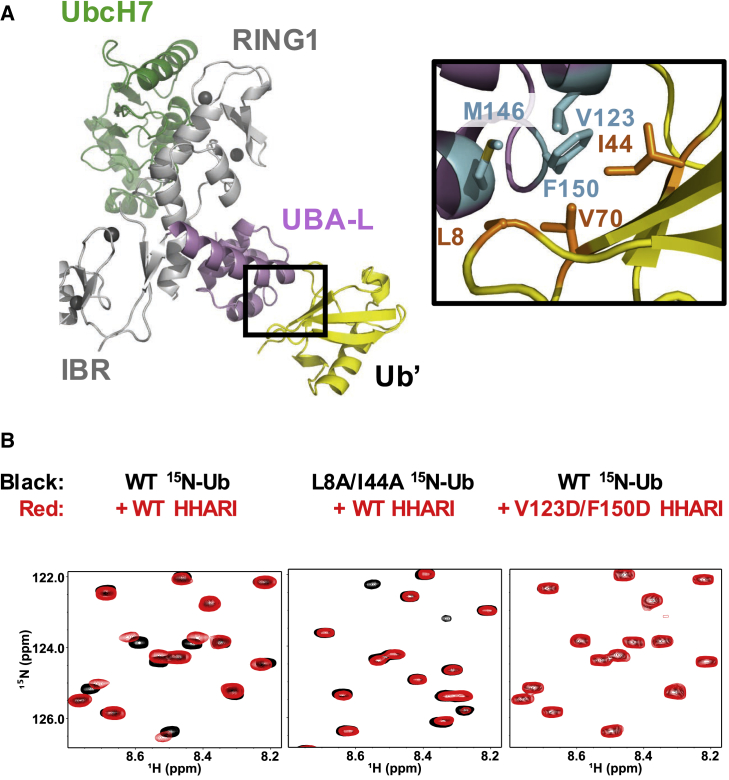
HHARI UBA-L Domain Binds Ub (A) HHARI UBA-L contacts Ub from a neighboring molecule (denoted as Ub′) in the crystal structure. Inset: central residues of the UBA-L/Ub′ interface include Val123, Met146, and Phe150 (HHARI, cyan sticks) and Leu8, Ile44, and Val70 (Ub, orange sticks). (B) (Left) Overlay of ^1^H,^15^N-HSQC-type NMR spectra of wild-type (WT) Ub (black) and Ub in the presence of HHARI (red). Ub residues affected by HHARI binding are identified by shifting and broadening of red peaks relative to black peaks. (Middle) The spectrum of L8A/I44A Ub in the presence of wild-type (WT) HHARI exhibits smaller perturbations, which indicates reduced binding. (Right) The HHARI mutant V123D/F150D shows substantially reduced binding to WT Ub, suggesting that HHARI residues Val123 and Phe150 are critical for Ub binding. See also Figures S6 and S7.

Analysis of the HHARI/UbcH7∼Ub crystal structure revealed that Ub from the UbcH7∼Ub bound to HHARI contacts the UBA-L domain of an HHARI molecule in a neighboring symmetry unit (denoted with a prime, Figure 4A). The Ub/UBA-L interface is composed of the hydrophobic surface of Ub (including residues Leu8, Ile44, and Val70) and residues Val123, Met146, and Phe150 of the UBA-L. To test whether the UBA-L binds Ub in solution, we took advantage of the ability of NMR to detect weak binding by collecting ^1^H,^15^N-heteronuclear single-quantum coherence (HSQC)-type NMR spectra of ^15^N-Ub in the absence (black) and presence (red) of full-length HHARI (Figure 4B). Chemical-shift perturbations (CSPs) in the form of peak shifting and broadening are clearly visible, indicating a specific interaction between Ub and HHARI (Figures 4B and S6A). CSP mapping onto the Ub structure reveals a binding surface centered around the Ile44 hydrophobic patch (Figure S6B). Mutation of residues on that surface abrogates the observed binding, as shown in the NMR spectra of L8A/I44A-Ub in the absence and presence of HHARI (Figure 4B). The HHARI UBA-L double mutant V123D/F150D shows substantially reduced binding to Ub (Figure 4B), corroborating that the Ub/UBA-L′ contact site observed in the crystal is similar to the weak interaction in solution.

To assess a potential functional role for the observed Ub/UBA-L′ binding, we considered several possibilities. First, we asked whether the UBA-L could serve to recruit the E2∼Ub conjugate to HHARI. Our structure per se does not support this possibility, as the Ub bound to the UBA-L is almost 40 Å away from its position in the UbcH7∼Ub conjugate bound to RING1, thus requiring a large domain-domain rearrangement (Figure S6C). Consistent with this structure-based prediction, the HHARI UBA-L double mutation V123D/F150D that decreases binding to Ub does not influence auto-ubiquitination activity (Duda et al., 2013). Furthermore, we do not observe inhibition of HHARI's auto-ubiquitination activity by high concentrations of free Ub^74^ (Ub that cannot be conjugated due to deletion of two C-terminal Gly residues) to potentially compete with UbcH7∼Ub (Figure S7A, lanes 8–13). Second, we wondered whether Ub binding might serve to activate auto-inhibited HHARI. However, assays that involve auto-inhibited HHARI in the absence and presence of high concentrations of free Ub^74^ are indistinguishable (Figure S7A, lanes 2–7). Therefore, while we cannot yet rule out a possible role for Ub binding to the UBA-L, at present we have not observed an effect on the reactions required for auto-ubiquitination.

Although a functional significance for an HHARI UBA-L/Ub remains undefined, the UBA-L domain is implicated in binding to the Ub-like protein, NEDD8, via an interaction between HHARI and neddylated Cullins (Kelsall et al., 2013, Scott et al., 2016). NMR binding experiments carried out on ^15^N-NEDD8 in the absence (black) or presence (red) of WT HHARI confirm that HHARI also binds weakly to free NEDD8 in solution (Figure S7B). That both Ub and NEDD8 bind similarly to the UBA-L is not surprising, based on the high similarity between the two proteins on their hydrophobic surface (>90% of residues at UBA-L/Ub′ interface are conserved between Ub and NEDD8, Figure S7C). However, while binding to neddylated Cullins has been shown to activate HHARI E3 activity, neither NEDD8 nor Ub alone are thought to cause conformational changes in HHARI that lead to its activation (Figure S7A) (Kelsall et al., 2013, Scott et al., 2016).

## Discussion

**Figure 5:**
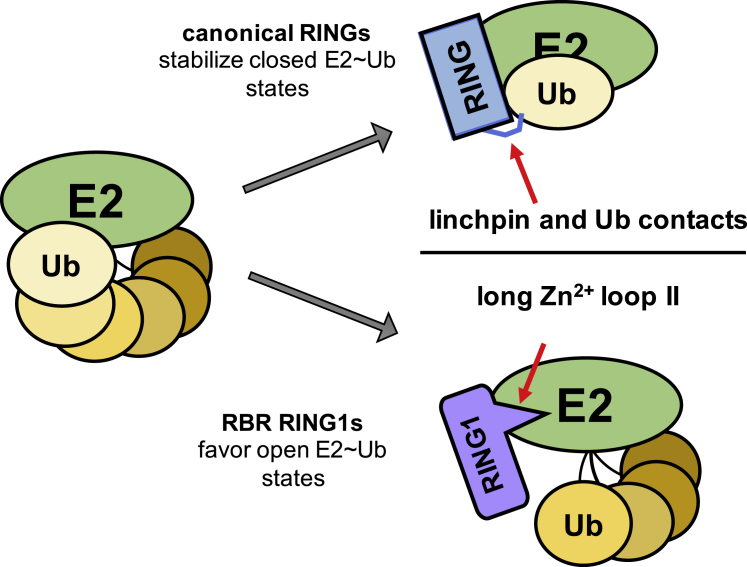
Model for Action of Canonical RING and RING1 Domains with E2∼Ubs (Left) E2∼Ub conjugates exist as dynamic ensembles of closed and open states whose populations vary depending on the E2 in question (Dove et al., 2016, Pruneda et al., 2011). (Upper right) Canonical RINGs stabilize closed E2∼Ubs via RING/Ub contacts and a linchpin interaction (red arrow) to activate transfer to amino groups. (Bottom right) RBR RING1s bind to an E2∼Ubs and the extended Zn^2+^-loop II serves as a wedge (red arrow) to inhibit adoption of closed states.

In this study, we set out to define how RBR E3s recognize and activate E2∼Ubs using HHARI and UbcH7∼Ub as an example, and discovered differences between canonical RING and RBR RING1 domains. Notably, an extension of Zn^2+^-loop II of HHARI RING1 is largely responsible for disrupting closed UbcH7∼Ub (Figure 1D). The loop tip contacts the crossover helix of UbcH7 on a surface known to stabilize closed E2∼Ub conjugates (Figure 3B) (Dou et al., 2012, Dou et al., 2013, Koliopoulos et al., 2016, Plechanovova et al., 2012, Pruneda et al., 2012, Scott et al., 2014). Indeed, a steric clash is observed upon modeling a closed E2∼Ub in place of UbcH7 (Figure S5C). We therefore propose that the distinct loop serves as a wedge that disfavors closed E2∼Ub states when bound to RING1 (Figure 5). Although we discovered this conformational restriction mechanism while studying UbcH7, an E2 that does not itself require inhibition of Lys reactivity, our findings, combined with previous ones, provide insight regarding how two highly similar domains, the RINGs and RING1s, can direct identical E2s such as UbcH5 to transfer Ub directly to Lys in the former case and only to Cys in the latter.

There are several functional explanations for why HHARI and (some) other RBR E3s have evolved to discourage bound E2∼Ub from populating closed states. First, it would prevent mistargeting by E2s such as UbcH5, Ubc13, and Ube2k that work with both RING and RBR-type E3s (Dove et al., 2016, Fiesel et al., 2014, Haddad et al., 2013, Kirisako et al., 2006, Lechtenberg et al., 2016, Lim et al., 2013, Stieglitz et al., 2013, Wenzel et al., 2011). We recently demonstrated that substituting HHARI's RING1 domain with a canonical RING domain that promotes the Lys-reactive closed state of UbcH5∼Ub generates a form of HHARI that no longer requires its active site to catalyze ligation by enabling Ub transfer to occur directly from the bound E2∼Ub to proximal Lys residues (Dove et al., 2016). Such off-mechanism activity would have deleterious consequences for an RBR E3, because bypassing the RBR active site leaves target selection and final product formation to a misactivated E2. Instead, an RBR E3 should dictate both its target selection and the product, as exemplified by the LUBAC complex (which contains HOIP [Kirisako et al., 2006]). HOIP mediates precise targeting by juxtaposing its catalytic RING2 and the N-terminus of an acceptor Ub (Stieglitz et al., 2013). This allows LUBAC to generate linear poly-Ub chains with high fidelity even when paired with the E2 Ube2K (also known as E2-25K), which would otherwise produce Lys-48-linked poly-Ub chains even in the absence of an E3. Maintaining the E2∼Ub in a state that can only undergo transthiolation ensures that Ub transfer proceeds through the RBR active site, thereby overriding any intrinsic preference of an E2 to build a particular type of Ub product or chain.

Some RBRs keep their activity in check through adoption of auto-inhibited states, yet we found that auto-inhibited HHARI can bind E2∼Ub. Thus, another functional advantage for the conformational restriction of bound E2∼Ub by RING1 is to inhibit non-productive discharge of Ub from highly reactive closed states until the E3 is activated and ready to accept the Ub onto its RING2 active site. A third explanation for RBRs favoring open E2∼Ub stems from our previous finding that the hydrophobic surface of the ∼Ub plays a role in recruiting HHARI RING2, as mutations on that surface greatly diminish formation of the E3∼Ub conjugate for HHARI and HOIP (Dove et al., 2016). This surface of Ub is sequestered in closed UbcH7∼Ub conformations (Dove et al., 2016), suggesting that even the non-Lys reactive E2 UbcH7 requires open E2∼Ub for productive Ub transfer. Although we did not observe a measurable difference in the formation of the E3∼Ub using WT-RING1-HHARI^RBR/H359A^ or the RING1 loop deletion mutant ΔHG-RING1-HHARI^RBR/H359A^ (Figure S2B), this could reflect either that E2∼Ub conjugates are highly dynamic ensembles despite “predominantly” populating an open or closed state (Dove et al., 2016, Pruneda et al., 2011), or the challenges of quantifying formation of the E3∼Ub thioester intermediate for HHARI. As the ΔHG-HHARI mutant does not completely inhibit the open UbcH7∼Ub state (as evidenced by NMR, Figure 1D), we conclude that the rate of the E2∼Ub open/closed transition is not rate limiting for the transthiolation reaction between UbcH7∼Ub and the HHARI active site under the conditions of our assay. Together with previously published results showing that mutations in the Ub hydrophobic surface affect the formation of E3∼Ub, the results suggest that recruitment of RING2 to the RING1-bound E2∼Ub is likely the rate-limiting step, at least under the conditions used in these studies.

Not all RBR E3s have an extended Zn^2+^-loop II (Figure 1B), and these may use alternative mechanisms to ensure that their bound E2∼Ub are open. For example, UbcH5∼Ub is bound to HOIP in an open state even though the short HOIP Zn^2+^-loop II does not contact UbcH5 crossover helix residues (Lechtenberg et al., 2016). In contrast to ∼Ub in our structure which appears not to contact its cognate HHARI, the analogous ∼Ub in the crystal structure of UbcH5∼Ub bound to HOIP-RBR-LDD contacts RING1 and the IBR, reminiscent of a HECT/E2∼Ub complex (Kamadurai et al., 2009, Lechtenberg et al., 2016). The HOIP-RBR-LDD/UbcH5∼Ub structure appears to represent a partially activated state with the E2∼Ub well positioned for Ub transfer, but the acceptor RING2 domain belongs to a different polypeptide due to a dimer swap. Therefore, details of the fully activated E2∼Ub/HOIP complex with its RING2 correctly positioned must await further studies. Nevertheless, as the transfer of Ub from the E2 to E3 active sites is a shared step in all RBR-catalyzed Ub transfer, presentation of an open E2∼Ub, either through the wedge mechanism defined here (RBRs with extended loops) or through additional interactions with the ∼Ub as seen in HOIP, is a unifying theme.

In sum, our study provides a structural explanation for the fundamentally different action of RING1s as compared with their eponymous cousins, the canonical RING domains. RING E3s couple E2∼Ub binding and activation in a single event that enables the E2∼Ub to “fire” directly onto substrate. By promoting an open E2∼Ub, RBR E3s inhibit E2∼Ub firing to allow formation of the E3∼Ub intermediate once the E3 is activated and its RING2 active site is exposed. This subtle difference in how an E2∼Ub is handled by an E3 is the deciding factor in whether the final Ub product attached to a substrate is dictated by the E2 or the E3. The activation step and subsequent transfer of Ub to a substrate must involve large-scale domain rearrangements in auto-inhibited RBR E3s. Details of how HHARI and other RBR E3s achieve these important final steps are undoubtedly the next structural mystery to be solved.

## STAR★Methods

### Key Resources Table

**Table:** 

REAGENT or RESOURCE	SOURCE	IDENTIFIER
Mouse monoclonal anti- GST antibody	Life Tein	LT0423
Mouse monoclonal anti-HA antibody	Life Tein	LT0422
Rabbit polyclonal anti-HA antibody	Bethyl Laboratories	A190-108A; RRID: AB_67465
Index HT	Hampton Research	HR2-134

**Bacterial and Virus Strains**		

ArcticExpress (DE3)RIL Competent cells	Stratagene	CAT# 230193
Rosetta (DE3) pLysS Competent cells	Millipore	CAT: 70956-3
One Shot BL21 Star (DE3)	Thermo Fisher Scientific	C601003
BL21-Gold (DE3) Competent cells	Agilent Technologies	CAT# 230132

**Chemicals, Peptides, and Recombinant Proteins**		

Phosphocreatine disodium salt hydrate	Sigma-Aldrich	P7936
Creatine Phosphokinase	Sigma-Aldrich	C3755
Adenosine 5'-triphosphate disodium salt hydrate	Sigma-Aldrich	A2383
Ammonium-15N chloride	Sigma-Aldrich	299251
IPTG	Gold Biotechnology	2481C100
PMSF	Sigma-Aldrich	P7626-100G
p-Aminobenzamidine-Agarose	Sigma-Aldrich	A8332
Thrombin from bovine plasma	Sigma Aldrich	T4648
Bovine erythrocyte Ubiquitin (for charging UbcH5a)	Sigma-Aldrich	79586-22-4

**Deposited Data**		

HHARI/UbcH7∼Ub structure	This paper	PDB: 5UDH

**Recombinant DNA**		

pet28n-UbcH7 (WT)	Wenzel et al., 2011	N/A
pet28n-UbcH7 (C86S)	Dove et al., 2016	N/A
pet28-UbcH7 (K96E, F63A)	This study	N/A
pGEX-2T-GST-Thrombin-HHARI-RING1-res177-270 (WT)	Dove et al., 2016	N/A
pGEX-2T-GST-Thrombin-HHARI-RING1-res177-270 (H234G; ΔH234/ΔG235)	This study	N/A
pGEX-2T-GST-Thrombin-HHARI-RBR-res177-395 (WT; I188A)	Wenzel et al., 2011	N/A
pGEX-2T-GST-Thrombin-HHARI-RBR-res177-395 (H395A)	Dove et al., 2016	N/A
pGEX-2T-GST-Thrombin-HHARI-RBR-res177-395 (D237R; Y215A; I188A/Y215A)	This study	N/A
pGEX-2T-GST-TEV-HHARI-FL (WT; F430A/E431A/E503A)	This study	N/A
pGEX4T1-TEV-HHARI-FL (V123D/F150D)	Duda et al., 2013	N/A
pGEX4T1-GST-Thrombin-UBCH7 (C86K)	Duda et al., 2013	N/A
pGEX4T1-GST-TEV-HHARI-res90-557	Scott et al., 2016	N/A
pET49b-HHARI-RING1 (res.184-286)	This paper	N/A
pGEX6P1-UbcH7	Stieglitz et al., 2012	N/A
pGEX6P1-UbcH5a	Stieglitz et al., 2012	N/A
pGEX6P1-UbcH7 (C17S/C86K, C137S)	This Study	N/A
pGEX6P1-UbcH5a (S22R, C85K)	Koliopoulos et al., 2016	N/A
pET28 mouse Uba1	Carvalho et al., 2012	Addgene, Plasmid #32534
pET21-HisTEV-Ubiquitin	This Study	N/A
pGEX4T1-GST-Thrombin-NEDD8	Goldenberg et al., 2004	N/A

**Software and Algorithms**		

NMRPipe/NMRDraw	Delaglio et al., 1995	https://spin.niddk.nih.gov/bax/software/NMRPipe/install/
NMRViewJ	Johnson and Blevins, 1994	http://www.onemoonscientific.com/nmrviewj
OriginLab Software for analysis of ITC data	Malvern	http://www.originlab.com
Wyatt ASTRA VI software		http://www.wyatt.com/products/software/astra.html
RAPD pipeline		https://github.com/RAPD
ShelxC/D/E	Sheldrick, 2010	
CRANK CCP4 pipeline	Ness et al., 2004	
COOT	Emsley et al., 2010	https://www2.mrc-lmb.cam.ac.uk/personal/pemsley/coot/
Refmac	Murshudov et al., 1997	
CNS	Brunger et al., 1998, Schroder et al., 2010	
Phenix	Adams et al., 2010	https://www.phenix-online.org/
XDS	Kabsch, 2010	
Molprobity	Chen et al., 2010	
PyMOL	Schrödinger, 2002	http://www.pymol.org/

### Contact for Reagent and Resource Sharing

Further information and requests for resources and reagents should be directed to and will be fulfilled by the Lead Contact, Rachel E. Klevit (klevit@uw.edu)

### Method Details

#### Protein Expression and Purification

All proteins described are full-length human sequences unless stated otherwise. Proteins were grown in LB or minimal M9 medium supplemented with [^15^N]-ammonium chloride using *Escherichia Coli* (BL21 DE3 cells) and induced with 200 μM IPTG at 16°C for 18-22 hours unless otherwise stated. Wheat and human E1 (Uba1) were lysed in 50 mM Tris-HCl pH 8.5, 1 mM EDTA, 1 mM DTT. Filtered (0.45μm) lysate was applied to Ubiquitin resin in the presence of 50 mM Tris-HCl pH 8.0, 2 mM ATP, 6 mM Phosphocreatine (Sigma-Aldrich) and small, varying amounts of Creatine Phosphokinase (Sigma-Aldrich) Kinase for 30min at room temperature. The resin was washed with 20 CV 50 mM Tris-HCl pH 8.0, 0.5 M KCl and E1 was elute with 50 mM Tris-HCl pH 8.0, 10 mM DTT. Elution was dialyzed into 25mM NaPi, 150mM NaCl, 1mM DTT, pH 7.0, concentrated, aliquoted and stored in 5% glycerol at -80°C.

Mouse His-Uba1 (pET28; a gift from C. Wolberger) was transformed into Bl21-Gold (DE3) competent cells and grown into Terrific Broth at 37°C and induced with 1 mM IPTG at OD_600_ = 0.6 at 18°C for 16 hours. Cells were lysed 50 mM Hepes pH 8.0, 300 mM NaCl, 20 mM Imidazole, 0.5 mM TCEP supplemented with 1 mM PMSF and additional protease inhibitors. The supernatant containing soluble Mouse His-Uba1 was applied to a Ni-NTA column; the column was washed extensively with 30-50 CV of 50 mM Hepes pH 8.0, 300 mM NaCl, 20 mM Imidazole, 0.5 mM TCEP and elution was performed with 50 mM Hepes pH 8.0, 300 mM NaCl, 300 mM Imidazole, 0.5 mM TCEP. The eluted fraction was concentrated to 5 mL and applied to a size exclusion column (XK16/60 Superdex S200) equilibrated in 25 mM Hepes pH 7.5, 150 mM NaCl, 10 mM MgCl_2_, 0.5 mM TCEP. The purified mouse His-Uba1 was concentrated to 100 μM, flash-frozen in liquid nitrogen, and stored at -80 °C.

His-ubiquitin (His-tag cleavable with TEV protease) was inserted into pET21. The plasmid was transformed into *E.Coli* Bl21-Gold (DE3) competent cells. Cells were grown in LB at 37°C until optical density (OD) reached a value of 0.6, at which point the temperature was lowered to 18°C and induction was performed by addition of 1mM IPTG. After 16 hours, cells were harvested by centrifugation at 4000 rpm for 30 minutes at 4°C, and re-suspended into 50 mM Hepes pH 8.0, 300 mM NaCl, 20 mM Imidazole, 0.5 mM TCEP, supplemented with 1 mM PMSF and two tablets of protease inhibitor per 50 mL of lysate. Lysis was performed by sonication followed by clarification by centrifugation at 20000 rpm for 45 minutes at 4°C. The supernatant containing soluble His-ubiquitin was applied to a pre-packed Ni-NTA column; the column was washed extensively with 30-50 column volumes of 50 mM Hepes pH 8.0, 300 mM NaCl, 20 mM Imidazole, 0.5 mM TCEP and elution was performed with 50 mM Hepes pH 8.0, 300 mM NaCl, 300 mM Imidazole, 0.5 mM TCEP. The eluted His-ubiquitin-containing fractions were concentrated to 5 mL and applied to a size exclusion column (XK16/60 Superdex S75) equilibrated in 25 mM Hepes pH 7.5, 150 mM NaCl, 10 mM MgCl2, 0.5 mM TCEP. Fractions containing pure His-ubiquitin were concentrated to 1 mM, flash-frozen in liquid nitrogen, and stored at -80 °C.

UbcH5a and UbcH7 (for ITC) were inserted into pGEX6-P1 that allows the production of N-terminally GST-fused proteins containing a 3C protease cleavage site between the GST-tag and the protein of interest. The plasmids were transformed into *E.Coli* Bl21-Gold (DE3) competent cells. Cells were grown into LB at 37°C until optical density reached a value of 0.6, at which point the temperature was lowered to 18°C and induction was performed by addition of 1 mM IPTG. After 16 hours, cells were harvested by centrifugation at 4000 rpm for 30 minutes at 4°C, and re-suspended into 50 mM Hepes pH 7.5, 150 mM NaCl, 0.5 mM TCEP supplemented with 1 mM PMSF and two tablets of protease inhibitor per every 50 mL of lysate. Lysis was performed by sonication followed by clarification by centrifugation at 20000 rpm for 45 minutes at 4°C. The supernatant containing soluble GST-E2 proteins was applied to a gravity column containing GST4B beads; columns were washed extensively with 30-50 column volumes of 50 mM Hepes pH 7.5, 150 mM NaCl, 0.5 mM TCEP. Cleavage of the GST-tag was performed on beads by addition of 3C protease and overnight incubation at 4°C with gentle agitation. Cleaved E2 proteins were concentrated to 5 mL and applied to a size exclusion column (XK16/60 Superdex S75) equilibrated in 25 mM Hepes pH 7.5, 150 mM NaCl, 0.5 mM TCEP. Pure E2 proteins were concentrated to 0.5 mM, flash-frozen in liquid nitrogen, and stored at -80 °C.

GST-NEDD8 (pGex4T1, a gift from N. Zheng, University of Washington) lysate was applied to GST resin (GE Healthcare) in 50 mM Tris-HCl, 200 mM NaCl, pH 8.0. GST was cleaved on the column with thrombin (Sigma Aldrich) at 37°C. Post thrombin capture (p-Aminobenzamidine-Agarose-Sigma Aldrich) size-exclusion chromatography (SEC) was performed in 25 mM NaPO_4_, 150 mM NaCl, pH 7.0. Ub, UbcH5, UbcH7, HHARI RING1 (aa 177-270), HHARI^RBR^ (aa 177-395), HHARI full-length were produced as described (Brzovic et al., 2003, Dove et al., 2016, Duda et al., 2013, Wenzel et al., 2011). All point mutations were introduced using the Quikchange mutagenesis kit.

HHARI (aa 90-557, HHARI^90-557^) used for crystallography was cloned as described (Scott et al., 2016) and purified by glutathione affinity chromatography, followed by TEV proteolysis to liberate GST, SEC, glutathione affinity chromatography to remove remaining GST/uncleaved fusion protein, and SEC in 25 mM Tris-HCl pH 7.6, 150 mM NaCl, 1 mM DTT. UbcH7^C86K^ was expressed similarly, but with dialysis during proteolysis with TEV and a final SEC step. His-Ub was purified by Ni^2+^ pulldown, followed by dialysis, size exclusion chromatography in 25 mM Tris-HCl pH 7.6, 150 mM NaCl, 1 mM DTT. Purified HHARI^90-557^ and UbcH7^C86K^-His-Ub used in crystallography were mixed 1:1 and concentrated to form a complex. The complex was purified by SEC in 25 mM Tris-HCl pH 7.6, 150 mM NaCl, 1 mM DTT.

#### Crystallization and Data Collection

Crystals of HHARI^90-557^/UbcH7^C86K^-His-Ub were grown with the hanging drop vapor diffusion method at room temperature. 2 μl drops contained 1 μl protein mixture (10-12 mg/ml protein in 25 mM Tris, pH 7.0, 150 mM NaCl, 1 mM DTT) and 1 μl well solution (7-10% PEG 5000 monomethyl ether (MME), 0.1 M HEPES pH 7.0, and 5% Tacsimate pH 7.0) over a 1ml reservoir volume. Crystals were flash frozen in 30% PEG 5000 MME, 0.1M HEPES 7.0, and 5% Tacsimate pH 7.0.

#### Structure Determination

Due to the limited diffraction quality of these crystals, a relatively poor molecular replacement solution was obtained with limited usefulness for initial model building. Therefore, de novo structure determination was pursued. To this end, there are 6 naturally occurring Zn clusters per HHARI protomer. Zn SAD data (peak, 1.2862 Å) was collected at the Advanced Photon Source NE-CAT Sector 24-IDC beamline and processed to 3.6 Å using the RAPD pipeline (https://github.com/RAPD). The structure was solved using ShelxC/D/E (Sheldrick, 2010) as implemented in the automated CRANK CCP4 pipeline (Ness et al., 2004). Autotracing efforts yielded limited results. Therefore, a difference anomalous map was generated for location of the zinc atoms and placement of the 2 HHARI moieties in the asymmetric unit (ASU) using our previously published HHARI structure (pdb 4KBL (Duda et al., 2013)). After manual placement of HHARI using COOT (Emsley et al., 2010), UbcH7 moieties were identified in the experimental map. UbcH7 pdb 5HPT (Zhang et al., 2016) was used for initial model building. The full ASU was built without addition of the relatively weak Ub moieties. Furthermore, significant sub-domain movements for HHARI and UbcH7 relative to the reference structures were apparent. Therefore, models were broken down into sub-domains for rigid body refinement with Refmac (Murshudov et al., 1997). Following rigid body refinement, the Ub portion of the complex was modeled using available structures in the pdb. Due to insufficient electron density, only one of the two Ubs could be confidently placed in the ASU corresponding to chain E of complex A/C/E. Although the second copy of the Ub was not included in refinement, superimposition of the two complexes based on HHARI and UbcH7 reveal a similar location for the Ubs in their respective larger complex. Next, the low-resolution DEN refinement protocol was implemented using CNS (Brunger et al., 1998, Schroder et al., 2010). Best results (model with lowest R_free_, reduced R_work_/R_free_ spread, and good Ramachandran statistics and best overall map quality) were obtained using the rigid body refined model as the reference model, a maximum likelihood target function, and DEN parameters γ 0.4 and κ 300. Phenix (Adams et al., 2010) was then used for successive rounds of positional and B-factor refinement. After many rounds of crystal optimization and screening of hundreds of crystals, data from a single crystal diffracting to 3.24 Å was obtained using the NE-CAT Sector 24-IDC beamline. The isomorphous data was integrated and scaled with XDS (Kabsch, 2010). Following rigid body refinement, the improved electron density maps allowed for further additions to the model, including loop regions and termini. Further refinement was carried out using Phenix and Refmac. The A/C/E complex is described throughout the manuscript unless otherwise noted. The final model includes HHARI chain A residues 98-162, 181-329, 339-392, 410-448, and 451-554; HHARI chain B 98-152, 184-328, 340-391, 409-441, and 457-552; UbcH7 chain C 1-151; UbcH7 chain D 1-153; Ub chain E 1-73. According to Molprobity (Chen et al., 2010), 96% and 0% of residues are within the favored and outlier regions of the Ramachandran plot, respectively. See Table 2 for pertinent data collection and refinement statistics. Structural cartoon figures were generated using PyMOL (Schrödinger, 2002).

#### Auto-ubiquitination Assays

The following proteins at the stated concentrations were combined and incubated at 37°C in 25 mM NaPO_4_, 150 mM NaCl, pH 7.0: 0.5 μM wheat E1, 20 μM Ub and either 0.5 μM, 1.5 μM, 4.5 μM UbcH7 GST-HHARI^RBR^ (Figure S1C) or 2 μM E2/GST-HHARI^RBR^ (Figure 3C, S4B, S4C, and S5A). Reactions were started by addition of 10 mM ATP and were quenched by addition of SDS-PAGE reducing buffer. Samples were run on SDS-PAGE gel, followed by western blots visualized using GST antibody (Life Tein, LT0423) against GST-HHARI^RBR^.

#### Generation of UbcH7∼HA-Ub for In Vitro Assays

6 μM human E1 (Uba1), 390 μM HA-Ub and 130 μM UbcH7 were incubated in 25 mM NaPO_4_ at 37°C with 10 mM ATP/MgCl_2_ for 60 min. UbcH7∼HA-Ub was subsequently purified by SEC at 4°C.

#### HHARI∼Ub Thioester Capture Assay

The HHARI^RBR^ (H359A) mutant has previously been shown to allow for trapping of the HHARI∼Ub (Dove et al., 2016, Duda et al., 2013). On ice, 10 μM preformed UbcH7∼HA-Ub was incubated with 10 μM of either WT-RING1-HHARI^RBR^ (H359A) or the RING1 loop deletion mutant ΔHG-RING1-HHARI^RBR^ (H359A) in 25 mM NaPO_4_, 150 mM NaCl, pH 7.0. A time point zero was taking immediately prior to addition of E3s and reactions were quenched with either reducing or non-reducing SDS-PAGE load dye. Samples were run on SDS-PAGE gel, followed by western blots, which were visualized using GST antibody (Life Tein, LT0423) against GST-HHARI^RBR^ and HA antibody (polyclonal, rabbit from Bethyl Laboratories, A190-108A) against HA-Ub.

#### UbcH7∼Ub Discharge Assay in Presence of Ub^74^

5 μM UbcH7∼HA-Ub was incubated with 2 μM full-length HHARI (WT or the activating HHARI mutant F430A/E431A/E503A (Duda et al., 2013)) in the presence of absence or presence of 100 μM free (untagged) Ub^74^ (a Ub mutant that contains Ub residues 1-74). A zero time point sample was taken immediately prior to addition of E3s. Reactions were performed at 37°C and quenched at given time points with either reducing or non-reducing SDS-PAGE load dye. Samples were run on SDS-PAGE gel, followed by western blots visualized using HA antibody (monoclonal, rabbit from Life Tein, LT0422) against HA-Ub.

#### Generation of Stable UbcH7∼Ubs

For crystallography, isopeptide-linked UbcH7(C86K)∼Ub was generated by incubating 150 μM UbcH7^C86K^, 15 μM Uba1, 450 μM His-Ub in 30 mM Tris pH 8.8, 50 mM NaCl, 5 mM ATP and 10 mM MgCl_2_ for ∼18 hours at 30°C. The resulting UbcH7∼Ub was purified by SEC in 25 mM Tris-HCl pH 7.6, 150 mM NaCl, 1 mM DTT. The UbcH7-C86S-Ub oxyester used for NMR studies was generated by incubating 10 μM human Uba1, 250 μM UbcH7(C86S), 750 μM Ub, 12.5 mM ATP at 37°C for 8 hours in 25 mM NaPO_4_, 150 mM NaCl, pH 7.0. E2∼Ub conjugates were purified by size exclusion chromatography (Dove et al., 2016).

For ITC, 400 μM UbcH7 (C17S, C86K, C137S) was incubated with non-cleavable His-tagged mouse Uba1, TEV-cleavable His-tagged Ub (200 μM) in 50 mM CHES pH 9.0, 150 mM NaCl, 10 mM MgCl_2_ and 10 mM ATP at 37°C for 16 hours. The final reaction was passed over Ni-NTA beads to remove uncharged UbcH7. His-Uba1, charged E2, and free His-Ub were eluted from the beads with 50 mM Hepes pH 8.0, 300 mM NaCl, 300 mM Imidazole, 0.5 mM TCEP. To cleave the His-tag from Ub, TEV protease was added at 4°C overnight in dialysis buffer containing 50 mM Hepes pH 8.0, 300 mM NaCl, 0.5 mM TCEP. His-Uba and un-cleaved His-tagged Ub were removed by Ni-NTA beads and finally UbcH7∼Ub was separated from free Ub by SEC (XK16/60 Superdex S75, equilibrated in 25 mM Hepes pH 7.5, 150 mM NaCl, 0.5 mM TCEP) to separate UbcH7∼Ub from free ubiquitin.

200 μM UbcH5a (S22R, C85K) was incubated together with non-cleavable His-tagged mouse His-Uba1 (1 μM), ubiquitin (300 μM) in 50 mM TRIS pH 10.0, 150 mM NaCl, 20 mM MgCl2 and 10 mM ATP at 30°C for 16 hours in a total volume of 2 mL. The reaction was applied to a size exclusion column (XK16/60 Superdex S75, equilibrated in 25 mM Hepes pH 7.5, 150 mM NaCl, 0.5 mM TCEP) to separate UbcH5a∼Ub from His-Uba1, uncharged E2 and free Ub. All samples were concentrated to 1 mM.

#### Isothermal Titration Calorimetry

ITC experiments were performed at 293 K using a Microcal iTC200 calorimeter (Malvern). Samples were prepared in a degassed buffer (25 mM Hepes pH 7.5, 150 mM NaCl, 0.5 mM TCEP). Cell solutions contained 25-30 μM E2 or E2∼Ub and syringe solutions contained of 250-300 μM RING1 constructs. For each titration, 20 injections of 2 μL were performed. Where possible, the integrated data, corrected for heats of dilution, were fitted using a nonlinear least-squares algorithm to a 1:1 binding curve, using the MicroCal Origin 7.0 software package. The fitting parameters are ΔH° (reaction enthalpy change in kcal·mol^-1^), K_b_ (equilibrium binding constant in M^-1^), and n (number of binding sites). Each experiment was repeated twice and average values are reported.

#### SEC-MALS

The HHARI/UbcH7 complex was prepared at 1.5mg/ml in 25 mM MOPS, 150 mM NaCl at pH 7.5 and placed in an auto sampler a 4°C. 25 μl sample volumes were injected onto a GE Superose 6 Increase 3.2/300 mm column (2.4ml) equilibrated with matched buffer. Experiments were conducted at room temperature (∼25°C) with a flow rate of 0.1ml/min. Absorbance at 280 nm, light scattering, and change in refractive index were measured with a GE AKTA Pure coupled to a Wyatt miniDAWN TREOS and Optilab T-rEX differential refractive index detector. Molar mass was calculated from the Raleigh ratio based on multi-angle (static) light scattering and protein concentration from the change in refractive index (dn/dc = 0.185). Analysis was performed using Wyatt ASTRA VI software. Finally, absorbance at 280 nm and molar mass distribution were plotted as a function of elution volume (ml) using Excel (Microsoft).

#### NMR Experiments

All NMR experiments were performed at 298 K in 25 mM NaPO_4_, 150 mM NaCl, pH 7.0, 10% D_2_O. UbcH7-O-Ub was used for increased stability. The following protein concentrations and field strengths were used for (^1^H,^15^N)-HSQC-TROSY experiments: Figure 1D (500 MHz): 50 μM ^15^N-Ub, 200 μM free UbcH7-O-^15^N-Ub, 160 μM UbcH7-O-^15^N-Ub + 200 μM HHARI RING1, 150 μM UbcH7-O-^15^N-Ub + 250 μM HHARI RING1 mutants (H234G or ΔH234G235); Figure 4B (800MHz): 50 μM ^15^N-Ub, 50 μM ^15^N-Ub + 20 μM of either WT or V123D/F150D-HHARI FL; 500 (MHz): 50 μM ^15^N-L8A/ I44A-Ub, 50 μM ^15^N-L8A/ I44A-Ub + 20 μM HHARI FL; Figure S7B (800 MHz): 50 μM ^15^N-NEDD8, 50 μM ^15^N-NEDD8 + 20 μM HHARI FL. NMRPipe/NMRDraw (Delaglio et al., 1995) was used to process NMR data. NMRViewJ (One Moon Scientific) was used for data visualization (Johnson and Blevins, 1994). The equation Δδ = [(Δδ^15^N/5)^2^ + (Δδ ^1^H)^2^]^1/2^ was used to calculated chemical shift perturbations of 2D HSQC-TROSY NMR experiments.

### Quantification and Statistical Analysis

For ITC, the integrated data, corrected for heats of dilution, were fitted using a nonlinear least-squares algorithm to a 1:1 binding curve, using the MicroCal Origin 7.0 software package. The fitting parameters are ΔH° (reaction enthalpy change in kcal·mol^-1^), K_b_ (equilibrium binding constant in M^-1^), and n (number of binding sites). Each experiment was repeated twice and average values are reported.

### Data and Software Availability

The structure of HHARI/UbcH7∼Ub complex has been deposited in the Protein Data Bank (PDB) under accession number 5UDH (see Key Resources Table).

## Author Contributions

K.K.D., K.R., L.M., D.M.D., J.L.O., B.A.S., and R.E.K. conceived the experiments and/or wrote the manuscript. K.K.D. performed the NMR. K.K.D. and X.S.W. ran auto-ubiquitination assays. L.M. collected all ITC measurements. K.H.R. aided in protein purification and provided crucial insight. D.M.D., J.L.O., and D.J.M. generated the isopeptide-linked UbcH7∼Ub/HHARI complex and performed the crystallography experiments.

## Supplemental Information

Document S1. Figures S1–S7

Document S2. Article plus Supplemental Information
