# Supplementary material for: Structural Studies of HHARI/UbcH7∼Ub Reveal Unique E2∼Ub Conformational Restriction by RBR RING1
Source: Structure. 2017 Jun 6;25(6):890–900.e5. doi: 10.1016/j.str.2017.04.013 (PMC5462532; doi:10.1016/j.str.2017.04.013)
Supplement: Document S1. Figures S1–S7 [file mmc1.pdf]

Structure, Volume *25*

Supplemental Information

Structural Studies of HHARI/UbcH7~Ub Reveal Unique E2~Ub Conformational Restriction by RBR RING1

Katja K. Dove, Jennifer L. Olszewski, Luigi Martino, David M. Duda, Xiaoli S. Wu, Darcie J. Miller, Katherine H. Reiter, Katrin Rittinger, Brenda A. Schulman, and Rachel E. Klevit

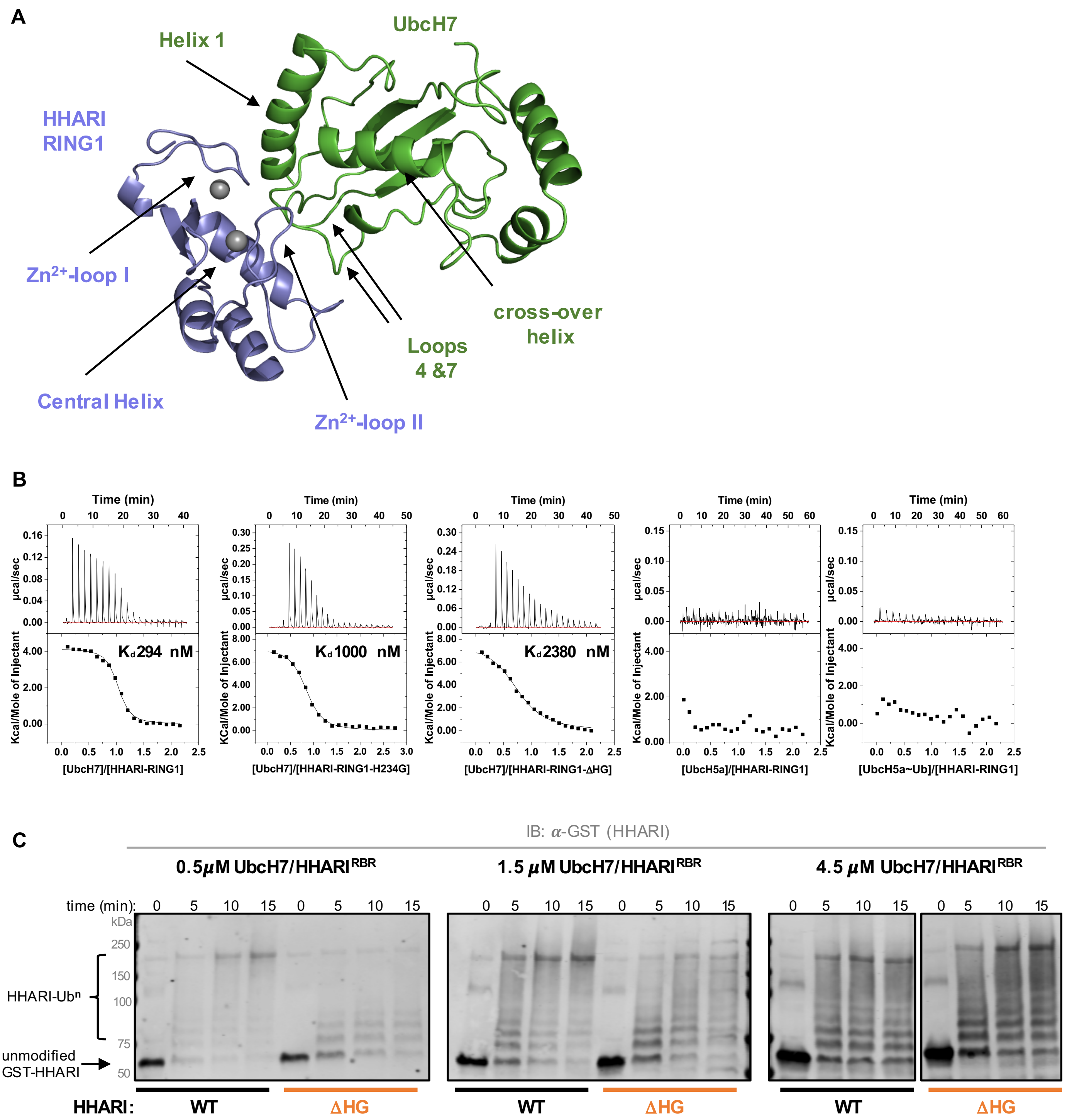

Figure S1. Related to Figure 1. A. Structural elements involved in the RING1/UbcH7 interface. Loops 4 and 7 and the N-terminus of helix 1 of UbcH7 and Zn^{2+}-loops I & II and the central helix of HHARI RING1 are identified. **B.** Calorimetric titrations of (from left to right) UbcH7 with HHARI RING1-wild-type, -H234G, or -ΔHG and UbcH5a or UbcH5a~Ub with HHARI RING1. Raw data and the normalized binding curve are presented and dissociation constants are reported when measurable. **C.** Auto-ubiquitination assays comparing activity of wild-type (WT) and loop deletion mutant (ΔHG) using GST-$HHARI^{RBR}$ that acts as both an E3 and a proxy substrate. This construct lacks the Ariadne domain and is therefore active (Duda et al., 2013). The assay was performed at three E2/E3 concentrations to examine potential affects of changes in affinity for the E2~Ub by the ΔHG mutant (K_D= 1.695μM) compared to WT HHARI (K_D=0.116μM). Times given are post-ATP addition and products were visualized by western blotting against GST. Images were cropped to remove molecular weight markers.

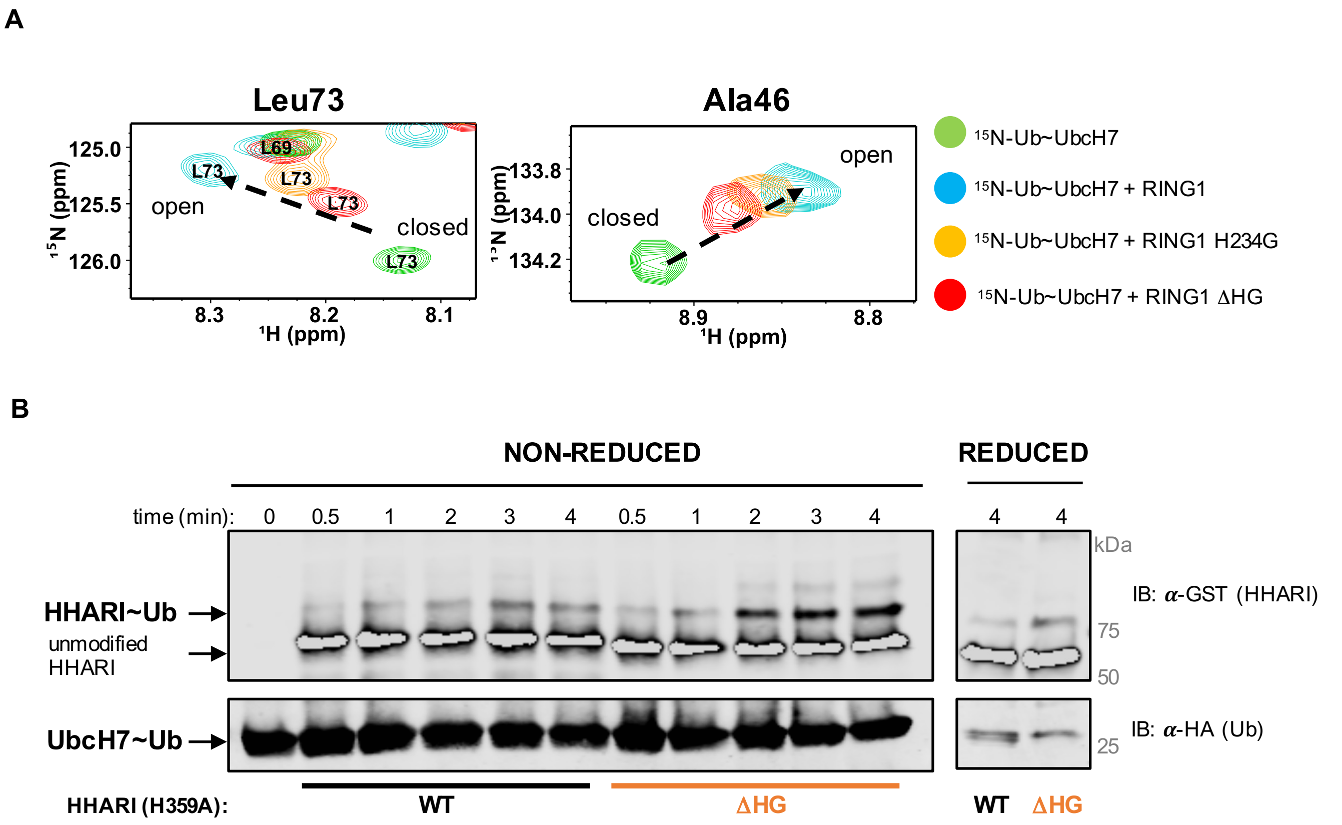

Figure S2. Related to Figure 1. A. In the absence of an E3, UbcH7~Ub populates closed states which are hallmarked by Chemical Shift Perturbations of affected residues (Leu73 & Ala46) in ^{15}N-Ub (green spectra). Binding of HHARI RING1 to UbcH7~Ub disrupts closed UbcH7~Ub (blue spectrum, Dove et al., 2016). Shortening of HHARI RING1 Zn^{2+}-loop II by two residues H234G235 (ΔHG) reduces the ability of HHARI RING1 to promote open UbcH7~Ubs (red spectrum), but H234G RING1 still promotes mostly open states of UbcH7~Ubs (orange spectrum). **B.** HHARI~Ub capture assay. The HHARI H359A mutation in proximity to the active site Cys has previously been shown to allow detection of the HHARI~Ub intermediate, which is typically short-lived (Dove et al., 2016; Duda et al., 2013). Preformed UbcH7~Ub (10 µM) was incubated with 10µM of either WT-RING1-HHARI^{RBR}(H359A) or the RING1 loop deletion mutant ΔHG-RING1-HHARI^{RBR}(H359A) and the formation of E3~Ub was monitored. Times given are post addition of the E3 to UbcH7~HA-Ub and products were visualized by western blotting against GST (HHARI) and HA (Ub). Images of the same Western blot were cropped to display each species separately.

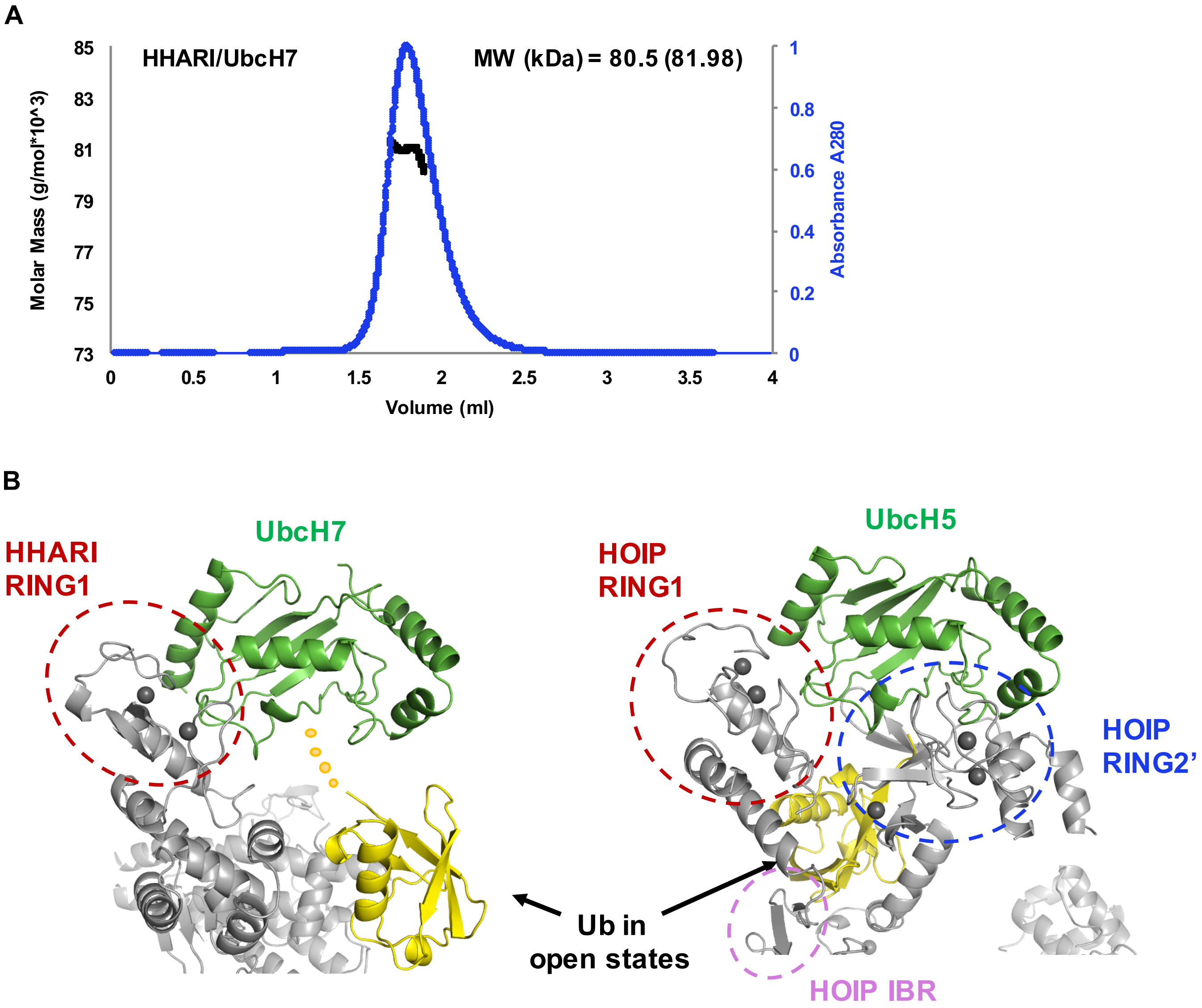

Figure S3. Related to Figure 2. A. Molecular weight (MW) of the HHARI/UbcH7 complex in solution was determined by SEC-MALS to be 80.5 kDa (theoretical value of dimer is given in parenthesis). The plot displays the molar mass distribution (black) superimposed with the chromatogram of absorbance at 280 nm (blue). **B.** Comparison of HHARI/UbcH7~Ub and HOIP-LDD/UbcH5~Ub**.** In both structures, the RING1 domain binds the E2 (green) and the E2~Ub is in an open conformation. *Left.* The Ub moiety (yellow) of UbcH7~Ub bound to HHARI does not contact any cognate HHARI domains. Yellow dots represent the C-terminus of Ub for which there is no observable density. *Right.* Ub of UbcH5~Ub bound to HOIP-LDD makes substantial contacts with the E3 via the C-terminal helix of RING1, the IBR, and RING2' of a neighboring polypeptide (PDB: 5EDV (Lechtenberg et al., 2016)).

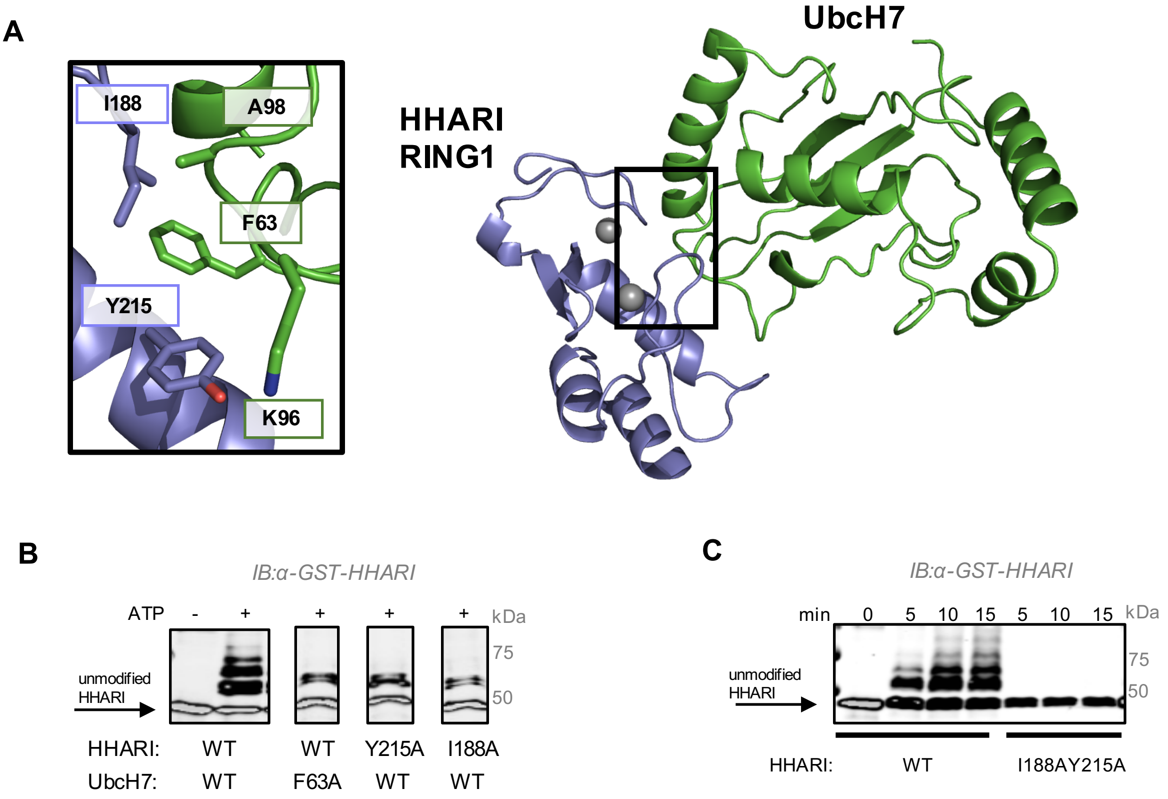

Figure S4. Related to Figure 3. **A.** Features of the RING1/UbcH7 interface that are reminiscent of canonical RING/E2 complexes. UbcH7 loop 4 residue Phe63 contacts HHARI RING1 residues Ile188 in Zn^{2+}-loop I and Tyr215 in the central helix. UbcH7 Phe63 is analogous to Phe62 in UbcH5 and RING1 Ile188 is analogous to a highly conserved Ile residue in canonical RINGs (for example, Ile26 in BRCA1) both of which play a pivotal role in canonical RING/E2 interactions; and hydrophobic residues emanating from the central helix of RINGs are also known to be involved in E2 interactions. **B.** Auto-ubiquitination assays with UbcH7 and GST-HHARI^{RBR} which acts as both E3 and proxy substrate. This construct lacks the Ariadne domain and is therefore active (Duda et al., 2013). Mutation of UbcH7 residue (F63A) or HHARI RING1 residues Y215A and I188A at the RING1/UbcH7 interface reduce auto-ubiquitination activity (Duda et al., 2013; Scott et al., 2016). Reactions were quenched with SDS-page load dye and products were visualized by western blotting against GST. Image was cropped to display 0 min (lane 1) and 15 min time points only (lanes 2 – 4). **C.** Auto-ubiquitination assays with UbcH7 and GST-HHARI^{RBR}. The double mutant I188A/Y215A of HHARI RING1 residues found at the RING1/UbcH7 interface drastically reduces auto-ubiquitination activity. Each indicated time post-ATP addition was quenched with SDS-page load dye and products were visualized by western blotting against GST. Images were cropped to removed molecular weight markers.

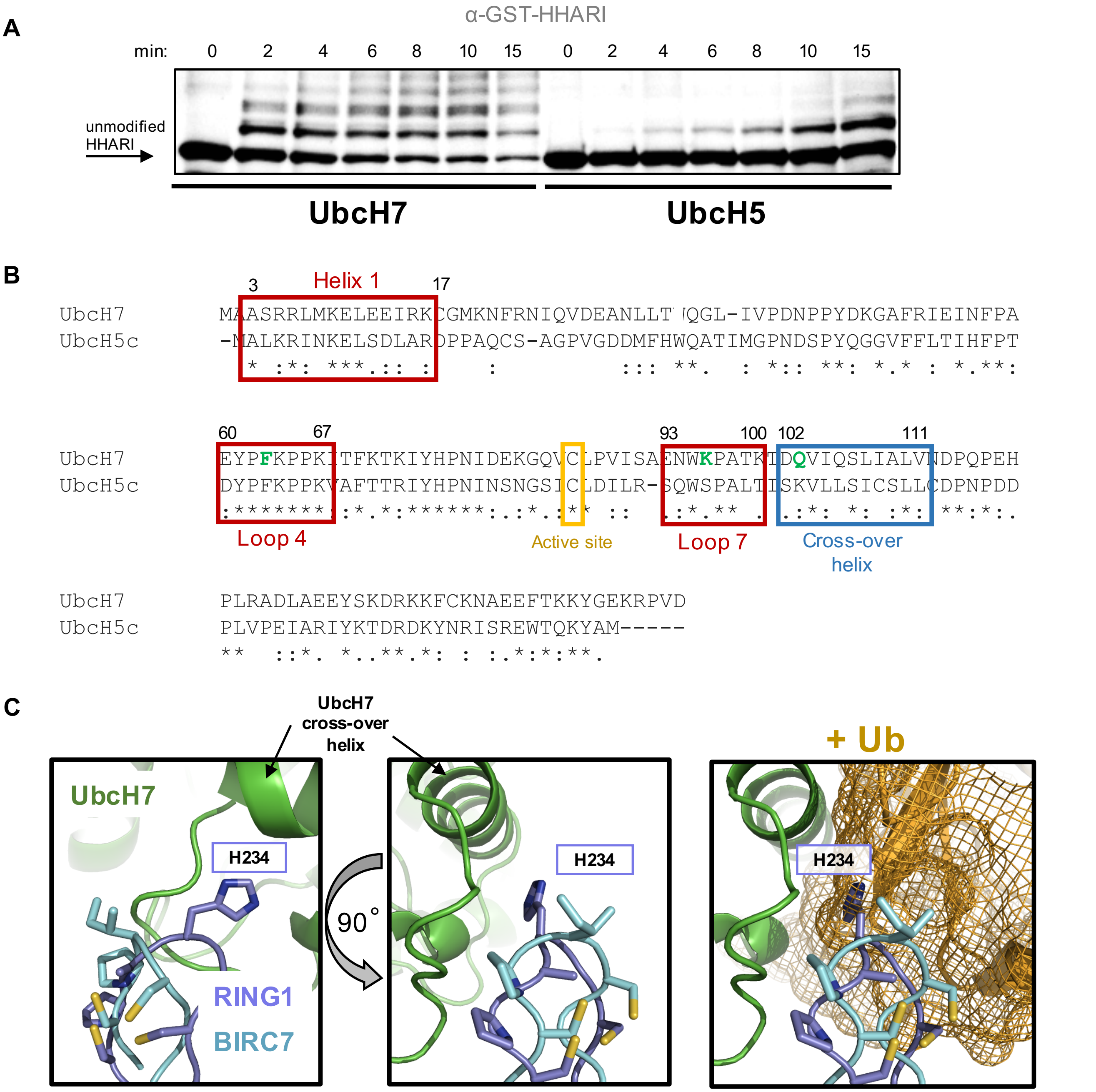

Figure S5. Related to Figure 3. Comparison of UbcH7 and UbcH5. **A.** Auto-ubiquitination assays comparing activity of UbcH7 and UbcH5 with GST-HHARI^{RBR} that acts as both an E3 and as proxy substrate. This construct lacks the Ariadne domain and is therefore active (Duda et al., 2013). Times given are post-ATP addition and products were visualized by western blotting against GST. **B.** Clustal Omega sequence alignment of UbcH7 and UbcH5c. Structural elements (Helix 1, Loops 4 & 7) typically involved in RING binding are marked with red boxes. Loop 4 is highly conserved between UbcH5c and UbcH7, while Helix 1 and Loop 7 are variable. Residues of the cross-over helix are indicated with a blue box. Active site Cys residues are highlighted by a yellow box. UbcH7 residues Phe63, Lys96, and Gln103 (highlighted in Fig. 3 and Fig. S4) are colored green. **C.** Structural overlays centered on the E2s of HHARI RING1/UbcH7~Ub and the canonical RING BIRC7/UbcH5~Ub. *Left & Middle*. Overlay of HHARI RING1 (purple) and BIRC7 (cyan) shows that Zn^{2+}-loop II of HHARI RING1 (with His234 at its tip), but not BIRC7 reaches toward the cross-over helix of the E2 UbcH7 (UbcH5~Ub bound to BIRC7 not shown). *Right*. Same view as in the middle panel with the Ub moiety from the closed state of UbcH5~Ub bound to BIRC7 displayed in yellow mesh. The structural overlay reveals that the tip of HHARI Zn^{2+}-loop II, C^{231}-P-A-H-G-C^{236}, would clash sterically with Ub in the closed state.

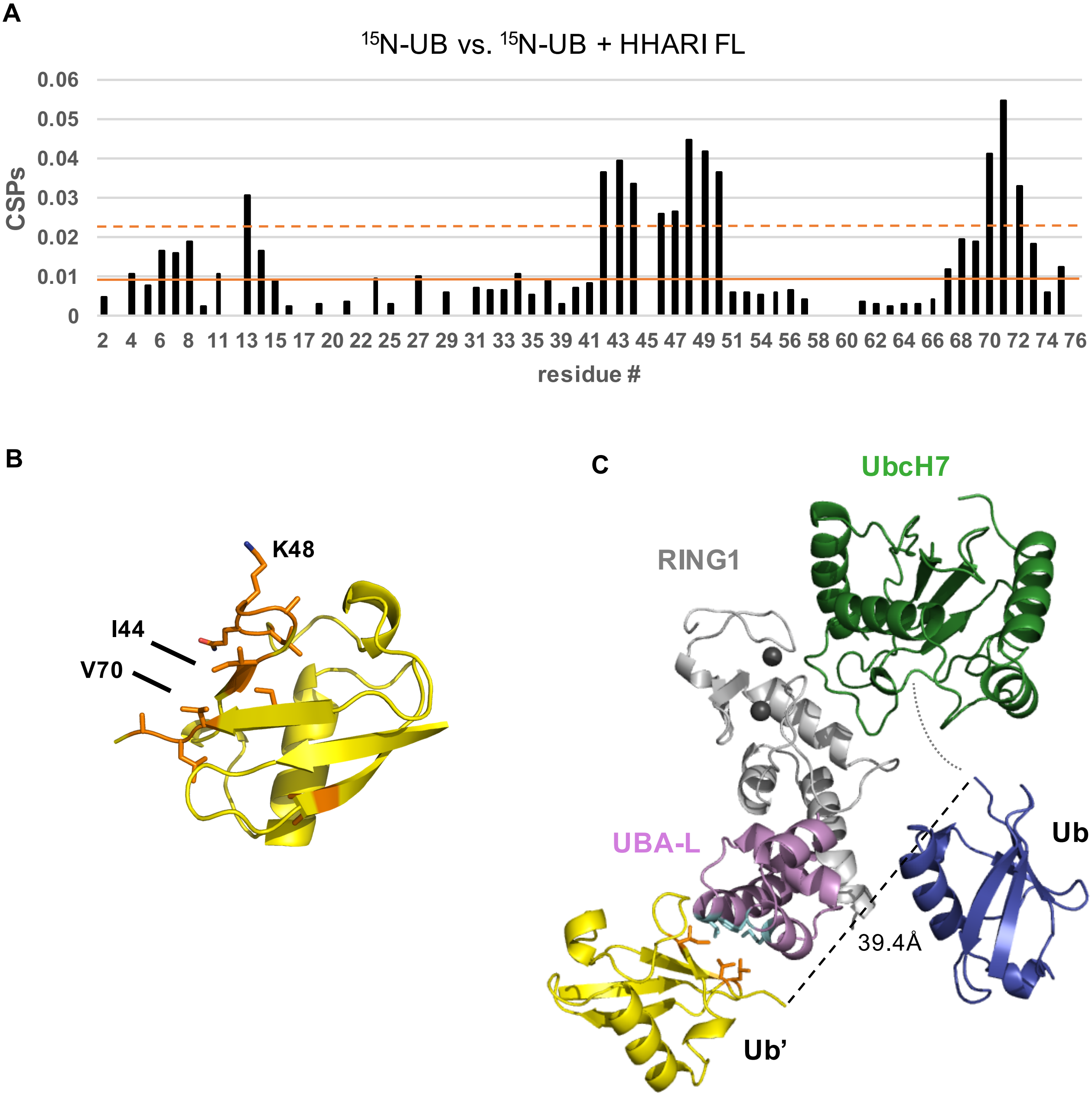

Figure S6. Related to Figure 4. HHARI binds Ub and NEDD8. **A.** Histogram of Chemical Shift Perturbations (CSPs) between ^{15}N-Ub in the absence and presence of 0.4 mole equiv. HHARI full-length (FL). The average (0.012) and AVG+1STDV (0.025) are indicated with a solid and dashed orange line, respectively. **B.** CSPs for wild-type Ub bound to HHARI-FL (Fig. 4B) are mapped onto a Ub structure (PDB 1UBQ). Ub residues with CSPs larger than 1STDV are colored orange. **C.** HHARI UBA-L binds Ub from a neighboring molecule (denoted as Ub’) in the crystal structure, but the distance of the Ub moiety of UbcH7~Ub to the UBA of the HHARI molecule to which UbcH7~Ub is bound is too far for Ub to bind to the UBA-L in *cis*. The distance between the C-terminal residue Leu73 of the conjugated Ub moeity (dark blue) and Leu73 or Ub’ (yellow) bound to the UBA-L is 39.4 Å as indicated. Residues represented as cyan sticks (UBA-L) and orange sticks (Ub’) are cntral residues of the UBA-L/Ub’ interface as shown in Fig. 4A.

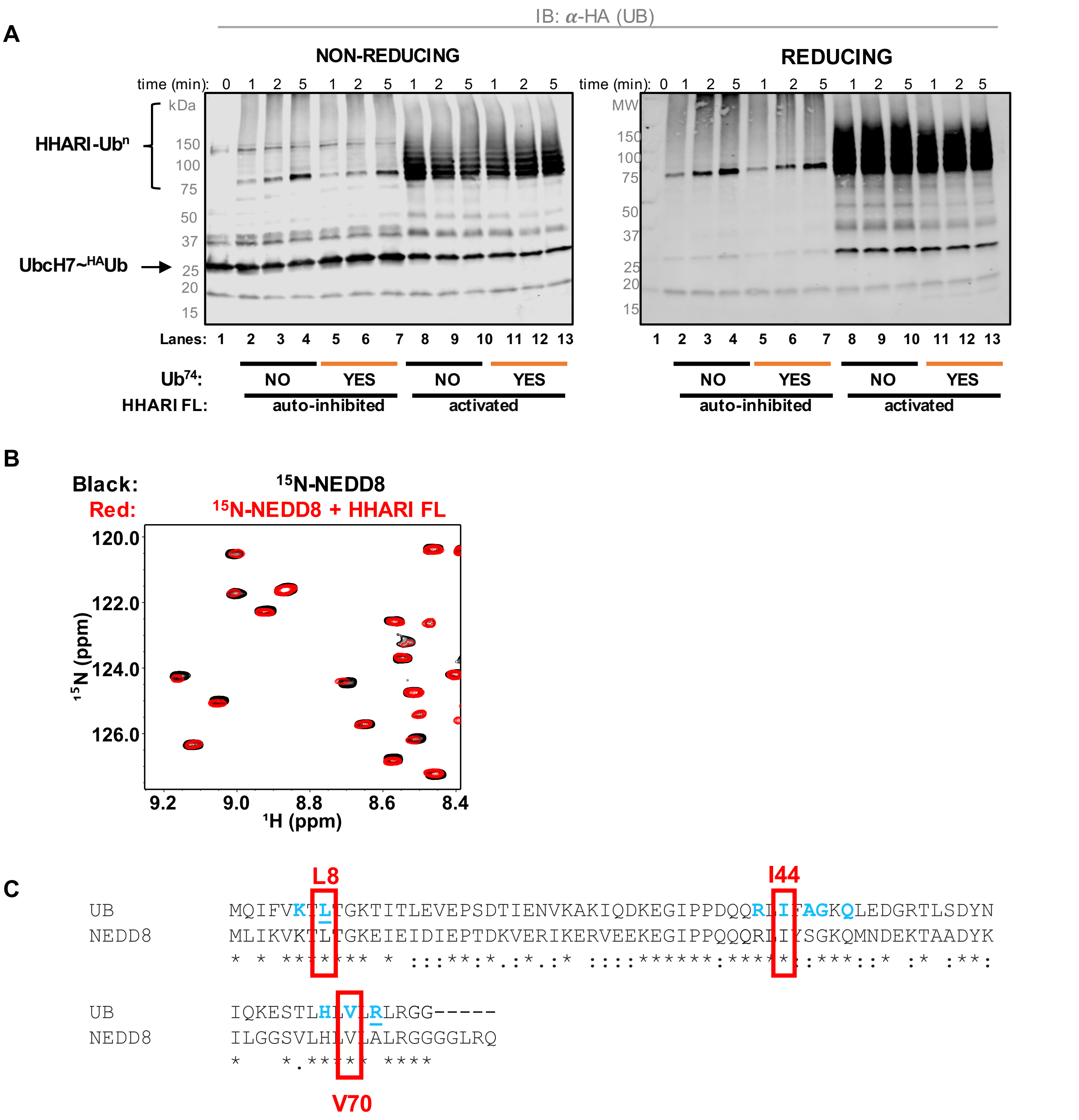

Figure S7. Related to Figure 4. A. Preformed UbcH7~HA-Ub was incubated with two different full-length (FL) HHARI constructs: wild-type (autoinhibited) or the activated mutant (F430A/E431A/E503A, (Duda et al., 2013)). Each reaction was performed in the absence or presence of free (untagged) Ub^{74}, a mutant that cannot be conjugated to E2 due to the lack of the last two Gly residues. Free (untagged) Ub^{74} does not allosterically activate either auto-inhibited or activated HHARI. Times given are post addition of E3 to UbcH7~HA-Ub and Ub transfer was monitored by western blotting against HA. Image was cropped to remove molecular weight markers. **B.** Overlay of ^{1}H-^{15}N-HSQC NMR spectra of free NEDD8 in the absence (black) and presence (red) of 0.4 mol equiv. of HHARI FL. Peaks shift and broaden in the presence of HHARI indicative of HHARI binding to NEDD8. **C.** Clustal Omega sequence alignment of Ub and NEDD8 reveals that UB residues (Leu8, Ile44 & Val70) central to HHARI UBA-L binding (Fig. 4 A&B) are conserved in NEDD8 (red boxes). Ub residues that contact a UBA-L of a neighboring HHARI subunit are highlighted in blue; of those, Leu8 and Val70 lack observable side chain density (underlined).
